# Spectrum of Biliary and Nonbiliary Neoplasms Growing and Spreading Within the Lumen of the Bile Ducts

**DOI:** 10.3390/cancers18091356

**Published:** 2026-04-24

**Authors:** Yasuni Nakanuma, Yasunori Sato, Yuko Kakuda, Takuma Oishi

**Affiliations:** 1 Department of Diagnostic Pathology, Shizuoka Cancer Center, Shizuoka 411-8777, Japan; y.kakuda@scchr.jp (Y.K.); ta.oishi@scchr.jp (T.O.); 2 Department of Diagnostic Pathology, Fukui Prefecture Saiseikai Hospital, Fukui 918-8503, Japan; 3 Department of Human Pathology, Kanazawa University Graduate School of Medicine, Kanazawa 920-8640, Japan; sato-ya@med.kanazawa-u.ac.jp

**Keywords:** bile duct, intraductal growth, precursors, cholangiocarcinoma, intraepithelial spread, cast-like neoplasm, cancerization of ducts, implantation, bile duct tumor thrombus

## Abstract

In the hepatobiliary system, the majority of neoplasms grow within the hepatic parenchyma; however, some arise, grow, and/or spread within the lumen of the intrahepatic large bile ducts and the perihilar/distal bile ducts (collectively referred to as large bile ducts), representing specialized ductal organs associated with unique peribiliary glands and being distinct from the intrahepatic small bile ducts and bile ductules embedded within the hepatic parenchyma. Precursors of cholangiocarcinoma (CCA) arising within the lumen of large bile ducts have recently been proposed. Neoplasms growing and spreading within the lumen of large bile ducts have been categorized into four groups and are discussed here in light of updated pathological findings. (i) Precursor(s) of CCA arising in the large bile ducts (large-duct-type intrahepatic CCA and perihilar/distal CCA): These precursors include high-grade biliary intraepithelial neoplasia (BilIN), intraductal papillary neoplasm of the bile duct (IPNB), and intraductal oncocytic papillary neoplasm (IOPN). High-grade BilIN presents as a flat, microscopic lesion with dysplastic cytoarchitectural alterations and grows along the luminal surface of large bile ducts, whereas the latter two present as grossly visible polypoid or tumorous lesions composed of papillary, villous, or tubular proliferation of neoplastic epithelium with delicate fibrovascular cores. These lesions may eventually progress to invasive CCA. Intraductal tubulopapillary neoplasm of the bile duct (ITPN), previously categorized as another precursor of CCA arising in large bile ducts, appears to represent a heterogeneous group of neoplasms with respect to progression and presumed cell of origin. Some ITPNs are frequently associated with nodular invasive carcinoma resembling small-duct-type intrahepatic CCA (SD-iCCA) and share genetic alterations with SD-iCCA; such cases may arise in association with small bile ducts or bile ductules. In contrast, other ITPNs exhibit cystic changes with tubulopapillary features and may arise in association with peribiliary glands or cysts. (ii) Secondary growth and spread of biliary neoplasms: This category comprises several patterns. First, intraepithelial neoplastic spread directly and continuously from the primary neoplastic lesion is observed in almost all cases of high-grade BilIN, IPNB, and IOPN; it spreads laterally along the luminal surface of the proximal and distal bile ducts and extends vertically into the adjacent peribiliary glands. Intraluminal cast-like spread in the bile ducts adjacent to the primary neoplastic lesion also occurs in some precursor lesions, particularly in ITPN. Implantation of a biliary neoplasm from one part of the biliary tract to another results in discontinuous, multifocal biliary neoplasms, particularly in IPNB, and occurs mainly in the distal bile ducts relative to the main tumor. Multicentric tumorigenesis may contribute to the multifocal development of precursors and CCA in the bile ducts. The accumulation of additional genetic alterations, beyond the common mutations detected in primary tumors, may contribute to metachronous recurrence of CCA after curative resection of the primary biliary tumor. Cancerization of the duct (COD) by CCA may also contribute to secondary growth and spread within the bile duct lumen. Specifically, flat-type cancerization of pre-existing non-neoplastic bile ducts, resembling high-grade BilIN, occurs in approximately one-third of hilar CCA cases. Intraductal polypoid, cast-like cancerization within the lumen of adjacent bile ducts, resembling polypoid precursors of CCA, can also occur in approximately one-tenth of SD-iCCA. (iii) Prominent intraductal polypoid growth of invasive CCA: Invasive CCA rarely presents with predominant intraductal polypoid carcinoma that is continuous with periductal infiltrating CCA; this pattern can be referred to as polypoid invasive CCA. (iv) Nonbiliary neoplasms presenting bile duct tumor thrombus (BDTT): BDTT associated with hepatocellular carcinoma and with extrahepatic malignancies extending into the bile duct lumen can mimic the intraluminal growth and spread patterns of the above-mentioned biliary neoplasms. In conclusion, intraluminally growing biliary neoplasms in the large bile ducts comprise a heterogeneous group that can be reasonably classified into four categories. This categorization may facilitate understanding of these intrabiliary growing neoplasms.

## Simple Summary

Several kinds of neoplasms arise, grow, and/or spread within the lumen of the intrahepatic large bile ducts and the perihilar/distal bile ducts (collectively referred to as large bile ducts): (i) Precursor(s) of cholangiocarcinoma (CCA) arising in the large bile ducts; (ii) secondary growth and spread of biliary neoplasms in addition to the primary neoplastic growth site; (iii) prominent intraductal polypoid growth of invasive CCA; (iv) nonbiliary neoplasms presenting bile duct tumor thrombus (BDTT) and mimicking primary intrabiliary growing neoplasms. Intraluminally growing biliary neoplasms in the large bile ducts do not represent a single entity but rather comprise a heterogeneous group that can be reasonably classified into four categories. Careful examination and evaluation of intraluminal growth and spread based on these categories could contribute to the development of a new field in biliary pathology and to novel therapeutic approaches for such neoplasms.

## 1. Introduction

Various types of neoplasms arise, grow, and/or spread in several anatomical compartments of the hepatobiliary system [1,2]. While the majority develop and/or grow within the hepatic parenchyma, some also arise and grow, either primarily or secondarily, and spread within the lumen of the intrahepatic large bile ducts and the perihilar/distal bile ducts (collectively referred to as large bile ducts) [1,2].

Recently, substantial progress has been made in the pathology of the latter lesions, including their terminology, and their molecular and genetic features are being increasingly clarified [1,3,4,5,6,7]. Accordingly, they can be subdivided into several categories. Representative intraductally growing biliary neoplasms include precursors of cholangiocarcinoma (CCA) arising in the large bile ducts—namely, large-duct-type intrahepatic CCA (LD-iCCA) and perihilar/distal CCA (p/d-CCA)—which are classified as high-grade biliary intraepithelial neoplasia (BilIN), intraductal papillary neoplasm of the bile duct (IPNB), intraductal oncocytic papillary neoplasm (IOPN), and intraductal tubulopapillary neoplasm of the bile duct (ITPN) [5]. Interestingly, similar or analogous neoplasms are known to develop in the pancreatic ducts, such as the intraductal precursors of pancreatic ductal adenocarcinoma (PDAC), which represent pancreatic counterparts of these biliary lesions [8,9,10,11,12,13].

In addition to primary neoplastic growth within the bile duct lumen, these neoplasms may also spread secondarily into the surrounding non-neoplastic bile ducts. Specifically, continuous intraepithelial extension along the bile duct directly from the primary growth site of precursor lesions [5,10] and implantation of biliary neoplasms (intrabiliary dissemination) [14] have also been reported. Multicentric tumorigenesis and metachronous recurrence may contribute to discontinuous spread or the development of intraluminal neoplasms [14,15,16]. Furthermore, cancerization of the duct (COD) by hilar CCA into adjoining non-neoplastic hilar bile ducts, as well as cancerization of small-duct-type intrahepatic CCA (SD-iCCA) into the intrahepatic large bile ducts, may result in polypoid neoplastic growth within the bile ducts [3,6,16]. These secondary patterns of spread confer distinct pathological and clinical features in addition to the primary growth of biliary neoplasms. Periductal infiltrating LD-iCCA and p/dCCA may also rarely present with predominant intraductal polypoid or cast-like growth in the affected bile ducts [17]. Bile duct tumor thrombus (BDTT) associated with hepatocellular carcinoma (HCC), as well as metastasis from extrahepatic malignancies extending into the bile duct lumen and resulting in BDTT, are occasionally encountered and can mimic the growth and spread patterns of primary biliary neoplasms within the bile duct lumen [2,18].

There are several hepatobiliary neoplasms which are not connected to the bile duct lumen, thus different from the above-mentioned neoplasms growing and spreading in the lumen of bile ducts [1,2,10]. Mucinous cystic neoplasm (MCN) is a representative one and is characterized by subepithelial ovarian-like stroma which are positive for estrogen and progesterone receptors and also for inhibin-α which are negative in the primary neoplasms in the lumen of bile duct [1,2]. In addition, the gallbladder and cystic duct are very close and anatomically continuous with the bile duct, and several types of neoplasms arise in their lumen [1,2]. However, in this review, MCN and the neoplasms growing in the cystic duct and gallbladder will not be discussed.

Herein, first, the unique anatomical features of the large bile ducts in the major biliary tract and the biliary neoplasms arising in these ducts will be discussed with reference to small intrahepatic bile ducts and bile ductules and to biliary neoplasms arising within the hepatic parenchyma, respectively. Second, intraluminal neoplasms arising, growing, and/or spreading in the large bile ducts are categorized into four groups (Table 1), and these four categories are subsequently reviewed in light of updated pathological findings. To date, such an approach to biliary neoplasms growing and spreading within the bile duct lumen has not been reported.

## 2. Two Categories of Biliary Neoplasms: Those Arising in the Large Biliary Tract Versus Those in the Hepatic Parenchyma

Various types of biliary neoplasms, or neoplasms with biliary phenotypes, arise, grow, and/or spread within the hepatic parenchyma as well as within the lumen of the intrahepatic large bile ducts, which are located in the major biliary tracts (i.e., larger portal tracts near and at the hepatic hilum and within the hepatoduodenal ligament) [1,2,5,9,10,14,19]. Typically, the former include SD-iCCA, whereas the latter include LD-iCCA and p/dCCA. These two types of biliary neoplasms exhibit distinct clinicopathological and molecular genetic features that reflect the two unique anatomical locations and structural characteristics of the hepatobiliary system [1,2,5,9,10,14,19].

### 2.1. Large Bile Ducts in the Large Biliary Tract Versus Intrahepatic Small Bile Ducts in the Hepatic Parenchyma

**Figure 1:**
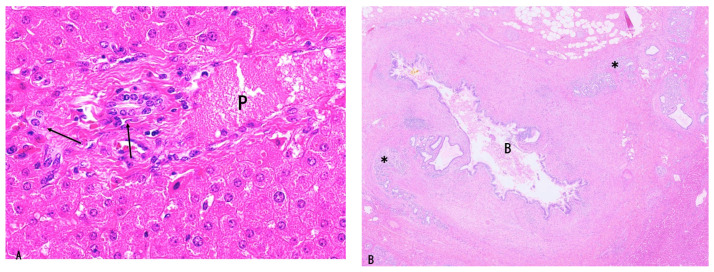
Small bile duct and large bile duct. (**A**): Small bile duct and bile ductules (arrows) in a small portal tract within the hepatic parenchyma. P, portal vein branch. ×200. Hematoxylin and eosin (H&E) staining. (**B**): Large bile duct (B) and peribiliary glands (*) in a large portal tract. ×50. H&E staining.

Bile ducts and their surrounding environments can be anatomically divided into two parts [20]: (i) intrahepatic small bile ducts located within the hepatic parenchyma (Figure 1A) and (ii) large bile ducts located in the major biliary tract (Figure 1B). The latter represent a specialized ductal organ with a unique periductal milieu in which peribiliary glands reside within loose connective tissue [10,20]. Peribiliary glands constitute a distinct glandular system annexed to the large bile ducts and drain into the lumen of these ducts through their own conduits [20]. These glands, which may contain foci of pancreatic exocrine acini [20], are also recognized as niches of pancreatobiliary stem cells [20,21,22]. In contrast, intrahepatic small bile ducts are components of smaller portal tracts embedded within the hepatic parenchyma and connect to hepatocytes via bile ductules or the canals of Hering, which are also regarded as niches of hepatic stem cells [20,23]. These two types of bile ducts differ in morphology and phenotype, including mucin expression profiles [20,24], and their principal features are summarized in Table 2. For example, diastase-periodic acid–Schiff (dPAS))-positive mucin is identified in the cytoplasm of large bile duct epithelial cells but is not detected in intrahepatic small bile ducts. MUC5AC, S100P, MUC1, and epithelial membrane antigen (EMA) are expressed in the cytoplasm of large bile duct epithelial cells. While the epitope of EMA is on the MUC1 mucin of the glycosylated form, EMA and MUC1 are examined separately in immunohistochemical study of epithelial cells and carcinoma cells [24]. In contrast, small bile ducts characteristically exhibit luminal expression of EMA and MUC1 and membranous expression of neural cell adhesion molecule (NCAM), whereas S100P expression is absent [24]. Nerve fibers and microvessels, including lymphatics, are denser in larger portal tracts than in smaller portal tracts. In addition, the peribiliary capillary plexus (PCP), derived from hepatic arterial branches, densely and regularly underlies the lining epithelium of large bile ducts; however, its distribution is sparse around small bile ducts and bile ductules [25].

The former may correspond to small cholangiocytes and the latter to large cholangiocytes, as recognized in cultured cholangiocytes from rats and mice [26,27,28]. These two types of cholangiocytes exhibit distinct morphologies and express different enzymes, as well as distinct molecular and gene expression profiles [26,27,28]. The anatomical and functional characteristics of these two types of bile ducts in the hepatobiliary system may be analogous to those of other organs composed of parenchyma and a draining ductal system. For example, in comparison with the kidney and urinary system, bile ductules and small bile ducts may correspond to proximal and distal renal tubules within the renal parenchyma, whereas large bile ducts may correspond to the renal pelvis and ureters. In the hepatobiliary system, the distinction between small and large bile ducts is not absolute, and there is a gradual transition between them. Septal bile ducts may represent an anatomical junction between intrahepatic small bile ducts and large bile ducts [20,24].

Recent studies have shown that the biliary tree contains two distinct anatomical regions that share similar phenotypes: (i) bile ductules and small bile ducts in and around smaller portal tracts and (ii) peribiliary glands, particularly serous glands, surrounding the large bile ducts [20,24,29,30]. Both regions are composed of cuboidal to low columnar simple epithelium and share similar histological and immunohistochemical profiles. Specifically, both are variably positive for MUC6, MUC1, IF6, D10, and TTF1 but negative for MUC5AC and MUC2 [20,24,29,30]. Interestingly, these anatomical regions are also recognized as niches for hepatic stem cells and pancreatobiliary stem cells, respectively [20,21,22,23]. The similarities between these two anatomical regions may help explain the distinctive tumorigenesis of several biliary neoplasms, such as bile duct adenoma [19,29,30], and possibly ITPN, as discussed below [7,11,12,13].

### 2.2. Biliary Neoplasms Arising and Growing in the Lumen of Large Bile Ducts Versus in the Hepatic Parenchyma

In 1997 and 2003, the Liver Cancer Study Group of Japan and Yamasaki proposed three macroscopic types of iCCA [31,32]: (i) mass-forming (MF) type, (ii) periductal-infiltrating (PI) type, and (iii) intraductal growth (IG) type. This macroscopic classification has since been standardized and is currently used internationally [1,3,6]. The MF type forms a well-defined, round mass located within the liver parenchyma. The PI type is characterized by a tumor that extends predominantly longitudinally along the large bile ducts, with frequent invasion into the hepatic parenchyma. In contrast, the IG type proliferates within the lumen of the bile ducts, presenting as a grossly visible papillary lesion or tumor thrombus and occasionally demonstrating superficial extension. More recently, iCCA has been subdivided into SD-iCCA and LD-iCCA based on anatomical location and distinct phenotypes [1,5,10]. Subsequent studies have shown that the MF type may correspond to SD-iCCA arising in the hepatic parenchyma, whereas the PI type corresponds to LD-iCCA arising in the intrahepatic large bile ducts [5,10]. It has also been demonstrated that the IG type does not represent a single biliary entity but rather comprises a heterogeneous group that includes several intraductally growing neoplasms [3,5,6,10].

Numerous biliary neoplasms arise and grow in the hepatic parenchyma as well as in the lumen of the large biliary tract. Interestingly, neoplasms arising and growing in the former share molecular and genetic features with HCC or combined hepatocellular–cholangiocarcinoma [33,34], in addition to exhibiting biliary morphology and phenotype. In contrast, biliary neoplasms arising and growing in the latter [5,9,10] share morphological and phenotypic features with duct-derived neoplasms, including pancreatic precursor lesions, in addition to their biliary phenotype [5,9,10,35]. Hepatic stem cells located in bile ductules or the canals of Hering within the hepatic parenchyma, as well as pancreatobiliary stem cells in the peribiliary glands, may contribute to the distinct characteristics and phenotypes of these two categories of biliary neoplasms, respectively [36,37]. CCA arising from large bile ducts develops through a noninvasive precursor–invasive sequence, and high-grade BilIN, IPNB, IOPN, and ITPN have been proposed as direct precursors of these CCAs [5,10]. In contrast, the precursors of biliary neoplasms arising in the hepatic parenchyma remain speculative and have not yet been identified [1,19].

Herein, neoplasms arising, growing, and/or spreading within the lumen of large bile ducts are discussed, and the IG-type of CCA is attempted to be refined and clarified in the light of recent progress in intraductal neoplasms [1,3,5,6,7,10,14].

## 3. Neoplastic Growth and Spread in the Lumen of Large Bile Duct

Herein, intraductally growing and spreading neoplasms within the lumen of large bile ducts are categorized into the following four groups (Table 1).

### 3.1. Precursors of LD-iCCA and p/d-CCA

**Figure 2:**
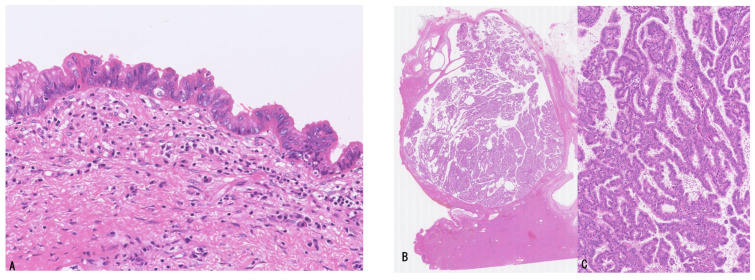

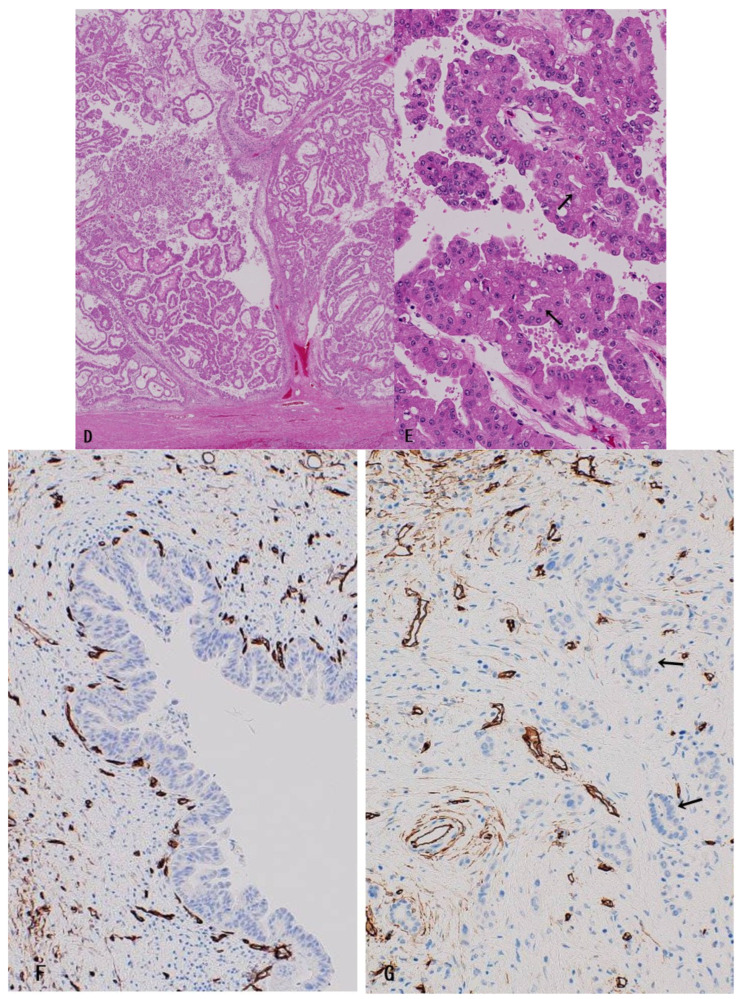
Precursors of cholangiocarcinoma. (**A**): High-grade biliary intraepithelial neoplasm (BilIN). Pseudostratified dysplastic biliary epithelium with mild loss of polarity. ×150. Hematoxylin and eosin (H&E) staining. (**B**): Intraductal papillary neoplasm of the bile duct (IPNB). A papillary neoplastic lesion without stromal invasion is present in a dilated intrahepatic large bile duct. Gastric subtype. Loupe figure. H&E staining. (**C**): The papillary structures show single-layered neoplastic epithelium with delicate fibrovascular cores. Gastric subtype. ×100. Higher magnification of (**B**). H&E staining. (**D**) Intraductal oncocytic papillary neoplasm of bile duct (IOPN) showing papillary neoplasm with fine fibrovascular stalk showing edematous change. ×100. H&E staining. (**E**) Neoplastic cells with acidophilic cytoplasm and hyperchromatic nuclei showing secondary lumina (arrows). ×150. Higher magnification of (**D**). H&E staining. (**F**) High-grade BilIN underlined by regular and dense peribiliary capillary plexus (PCP). ×150. CD34 immunostaining and hematoxylin. (**G**) Invasive tubular cholangiocarcinoma (arrows) embedded in fibrous tissue but not underlined by PCP. ×150. CD34 immunostaining and hematoxylin.

The majority of LD-iCCA and p/dCCA are considered to derive from several precursor lesions identifiable on the luminal surface of, and also within the peribiliary glands of, the large bile ducts through stepwise tumorigenesis and tumor progression [1,5,9,10]. These precursors differ from those of SD-iCCA [1,19,38,39]. Based on recent studies, the following precursor lesions have been proposed: (i) flat and microscopic precursors (high-grade BilIN) (Figure 2A) [1,5,10] and (ii) grossly visible papillary or polypoid/tumoral precursors (IPNB, IOPN, and ITPN) (Figure 2B–E) [1,5,10,11,12,13,14,40,41]. These precursors may be classified as the IG type according to the macroscopic classification of iCCA [31,32], although this classification was established before the recognition of these precursor lesions.

Differentiation of these precursors from reactive hyperplastic lesions of bile ducts is important for practical diagnostic pathologists [2,5,10,41]. In addition to immunohistochemical markers such as S100P which was diffusely and strongly expressed in high-grade precursors but focal or weak in reactive lesions [10], cell kinetic profile such as Ki-67 index may also be useful in such differentiation [2,20]. In this review, the development and progression of precursors will be mainly discussed rather than practical differentiation of these precursors from reactive or hyperplastic lesions.

Grossly visible tumorous or polypoid lesions of IPNB and IOPN are regarded as representing the primary growth sites in the affected bile ducts where they arise. In contrast, high-grade BilIN is a flat or microscopic lesion characterized by superficial, homogeneous, and often extensive intraepithelial spread. Therefore, it is difficult to identify the primary growth site or earliest neoplastic focus of high-grade BilIN within the affected bile ducts. High-grade BilIN is typically observed spreading adjacent to invasive CCA [5,10]; thus, lesions immediately adjacent to invasive CCA or the central portion of superficially spreading high-grade BilIN may represent the primary growth site. High-grade BilIN, IPNB, and IOPN are considered to arise from the lining epithelium of the large bile ducts through stepwise tumorigenesis. However, several reports have suggested that some neoplastic lesions may originate in the peribiliary glands and subsequently spread into the bile duct lumen through their own conduits and grow in the bile duct lumen [5,9,10,42].

In contrast, the primary growth pattern or early lesions of ITPN appear to be heterogeneous [7,11,12,13,43,44]. Specifically, some ITPNs without invasion or with only minimal invasion present primary neoplastic growth within the lumen of the large bile ducts [7,11,12,13]. In contrast, others are associated with nodular parenchymal invasion or SD-iCCA [11,12,13], which may reflect extension of intraductal neoplastic growth into the large bile ducts (cancerization of the duct) from SD-iCCA [7]. Additionally, some ITPNs with cystic and tubulopapillary patterns may involve the peribiliary glands and cysts [10,43,44]. Therefore, ITPN is discussed separately from IPNB and IOPN.

These precursor lesions exhibit characteristic growth at their site of origin (primary neoplastic growth) and almost invariably demonstrate intraepithelial neoplastic spread in the surrounding bile ducts (secondary intraepithelial spread) [5,10]. Herein, primary neoplastic growth and secondary intraepithelial spread are discussed separately.

#### 3.1.1. High-Grade BilIN

High-grade BilIN without stromal invasion, or with only minimal stromal invasion, is occasionally encountered in bile ducts affected by chronic, long-standing biliary diseases such as hepatolithiasis [1,5,10,45,46]. It is more frequently identified in bile ducts adjacent to invasive LD-iCCA and p/d-CCA, and more than 60% of these CCAs are associated with high-grade BilIN [5,10]. These findings suggest that the majority of these CCAs may arise from high-grade BilIN [5,10].

**Pathology**

High-grade BilIN is a preinvasive intraepithelial neoplasm characterized by a flat, pseudostratified, micropapillary, or pseudopapillary configuration with cellular and nuclear pleomorphism and loss of polarity and is appropriately designated as high-grade dysplasia or carcinoma in situ [1,5,10,45,46] (Figure 2A). This lesion spreads along the luminal surface of large bile ducts, involving a variable but often extensive area, and may be grossly recognized as discolored, velvety, granular, or rough mucosa, although it may also be macroscopically inconspicuous [5,10,45,46]. The lesion is also identifiable within the peribiliary glands of large bile ducts and their conduits [5,10,20]. Although the majority of high-grade BilINs belong to the biliary epithelial lineage, gastric and intestinal lineages are occasionally observed, whereas the oncocytic lineage is rare [5,10]. This lesion may correspond to high-grade dysplasia or carcinoma in situ identified at the bile duct margin in patients undergoing surgical resection for LD-iCCA or p/dCCA [47,48,49].

**Progression to invasive carcinoma**

High-grade BilIN may eventually progress to stromal invasion into the bile duct wall and periductal tissue, resulting in the development of the PI-type of iCCA [5,10,31,32]. The invasive carcinoma is more aggressive than high-grade BilIN confined to the luminal surface of the bile ducts [5,10], although the cellular lineage identified in the precursor lesions is generally retained in the invasive component [5,10].

Several types of microvasculature supplying neoplasms have been reported: newly formed blood vessels (neoangiogenesis), which constitute a component of the tumor microenvironment of invasive carcinoma, and pre-existing blood vessels that may be utilized by certain neoplasms as tumor-supplying vessels (vessel co-option) [25]. In the large bile ducts, the biliary lining epithelium and the underlying capillaries—namely, PCP derived from hepatic arterial branches—form a characteristic biliary epithelium–PCP alignment [25]. All cases of high-grade BilIN are underlain by regularly distributed PCP serving as supporting vessels through vessel co-option (Figure 2F), whereas p/d-CCA and LD-iCCA associated with high-grade BilIN are supplied predominantly by neoangiogenic vessels accompanied by fibrous stroma. The process of invasion is characterized by a reduction in PCP surrounding invasive carcinoma (Figure 2G) and the acquisition of neoangiogenesis, distinguishing it from high-grade BilIN [25]. Tumor budding, including features consistent with epithelial–mesenchymal transition, has also been reported at the early stage of stromal invasion [50,51].

**Molecular and genetic features**

High-grade BilIN is histologically well defined [5,10,45,46] but has not yet been systematically characterized at the molecular and genetic levels [52,53]. Similar to the pancreatic intraepithelial neoplasia (PanIN)–PDAC sequence model, LD-iCCA and p/d-CCA may also follow a stepwise carcinogenic process involving sequential molecular and genetic alterations through high-grade BilIN [10,45,46,52,53,54,55,56,57]. The accumulation of genetic alterations in high-grade BilIN—such as overexpression of the polycomb group protein EZH2, hypermethylation of the P16^INK4A^ promoter, increased expression of autophagy-related proteins, and decreased expression of p16—may be involved in the early phase of stromal invasion. In addition, p21 and cyclin D1 expression, along with downregulation of DPC4 and p16, have been observed during the histological progression of BilIN [10,55]. TP53 mutation, loss of SMAD4, and altered expression of glucose transporters may also contribute to the carcinogenesis of BilIN. In hepatolithiasis and associated CCA, *KRAS* mutations occur in approximately 33% of BilIN lesions associated with concomitant iCCA, and these mutations are considered early molecular events in BilIN progression, whereas *TP53* mutation represents a later molecular event. More recently, Loeffler et al. [52] demonstrated that clustering analysis of 49 deregulated microRNAs supported the concept of BilIN as a tumor precursor and identified miR-451a and miR-144-3p as putative tumor suppressors. Goeppert et al. [53] reported that expression of Deleted in Malignant Brain Tumors 1 (DMBT1), a known tumor suppressor, was increased in BilIN compared with normal tissue and invasive cholangiocarcinoma, suggesting that DMBT1 upregulation may play a role in early cholangiocarcinogenesis.

#### 3.1.2. IPNB and IOPN

IPNB and IOPN are grossly visible precursors of approximately 7% of LD-iCCA and p/d-CCA cases [5,10,58]. At the time of surgical resection, stromal invasion is identified in more than half of IPNB and IOPN cases [5,10,58], and such lesions are classified as IPNB or IOPN associated with invasive carcinoma [1,5,10,41,58].

**Pathology and phenotypes**

IPNBs and IOPNs present as grossly visible, fragile, tumorous or polypoid lesions within the lumen of dilated large bile ducts. The gross features of IPNB and IOPN depend on their anatomical location, the degree of mucin hypersecretion, and the presence of macroscopic invasion into the liver parenchyma [5,10,58]. IPNBs located in the intrahepatic bile ducts tend to be larger than those in the extrahepatic bile ducts and often exhibit marked ductal dilatation or multilocular cystic changes [5,10,58]. Interestingly, IPNBs and IOPNs arising in the peribiliary glands of large bile ducts may demonstrate diverticular or aneurysmal dilatation along the affected ducts [5,10,58].

Histologically, some lesions are predominantly villous or papillary, whereas others show predominantly papillotubular or tubular patterns lined by dysplastic cuboidal to low-columnar epithelium arranged in a single layer or in a pseudostratified manner, with fine fibrovascular stalks and well-capillarized vasculature (Figure 2B–E) [5,10,25,58].

***(1) IPNB***

IPNBs are classified into intestinal, gastric, and pancreatobiliary (PB) subtypes according to cellular lineage [5,10,58,59]. The intestinal subtype is composed of columnar cells with cigar-shaped nuclei and basophilic or amphophilic cytoplasm, exhibiting pseudostratified nuclei and diffuse immunohistochemical expression of CK20 and/or CDX2. The gastric subtype demonstrates papillary or tubular (glandular) neoplastic epithelium resembling gastric foveolar epithelium positive for MUC5AC and pyloric gland–type epithelium positive for MUC6 (Figure 2B,C). Some cases predominantly exhibit a foveolar pattern or a pyloric gland pattern, whereas others display both components in approximately equal proportions. The PB subtype shows single-layered small- to medium-sized cuboidal to low-columnar neoplastic epithelium with slightly acidophilic cytoplasm and centrally or basally located pseudostratified nuclei. The epithelial cells and nuclei resemble those of simple epithelium of the bile duct or pancreatic duct and form numerous delicate papillary structures.

***(2) IOPN***

IOPNs present single- to multilayered medium-sized cuboidal to low-columnar epithelium with eosinophilic, granular cytoplasm and frequent formation of secondary lumina [40,60] (Figure 2D,E). The epithelial cells contain numerous enlarged mitochondria, accounting for their oncocytic appearance. Arborizing papillary and/or cribriform structures are also observed. The stroma may be thin and fibrous and can additionally show edematous or inflammatory changes.

**Type 1 and type 2 subclassification of IPNB and IOPN**

IPNBs have traditionally been graded pathologically into low-grade and high-grade dysplasia according to cytoarchitectural alterations [1,5,10,58,61,62]. To complement this, onventional grading system, a type 1 and type 2 subclassification has been proposed [1,10,16,58,62]. This subclassification is based on two characteristic features of IPNB: (i) similarities to other grossly visible precursors of the pancreatobiliary system, such as prototypical intraductal papillary mucinous neoplasm (IPMN) and IOPN of the pancreas, as well as intestinal adenoma of the ampulla; and (ii) distinctive cytoarchitectural alterations observed in IPNB and IOPN. Type 1 IPNB (gastric and PB subtypes) shares many features with prototypical gastric and PB subtypes of pancreatic IPMN and with pancreatic IOPN, exhibiting relatively regular histology. Type 1 IPNB of the intestinal subtype similarly resembles low- and high-grade intestinal adenomas of the ampulla, respectively. In contrast, type 2 gastric and PB subtypes of IPNB and IOPN demonstrate variable differences from their pancreatic counterparts, and type 2 intestinal IPNB differs from intestinal adenoma of the ampulla and also from the gastric subtype of IPMN.

Approximately 40% of IPNBs reportedly belong to type 1, whereas the remaining 60% are classified as type 2 [58,62,63]. Mucin hypersecretion is more frequent in type 1 (61%) than in type 2 (37%). Type 2 IPNB is frequently associated with stromal invasion, the PB subtype and development within the extrahepatic bile duct compared with type 1 IPNB. Recent studies have shown that type 1 is associated with more favorable postoperative outcomes compared with type 2 [64], whereas traditional grading does not correlate with postoperative survival [58]. This novel pathological subclassification has recently been validated through an interobserver agreement study [65].

**Progression to invasive carcinoma**

Invasive carcinoma associated with IPNB and IOPN has been histologically classified into three patterns (A, B, and C) [66]. Pattern A is characterized by microscopic foci of invasive carcinoma within the fibrovascular stalks or confined to the bile duct mucosa and wall. Pattern B is defined by invasive carcinoma extending into the periductal connective tissue and adjacent organs, predominantly near or beneath the intraluminal components of IPNB, with invasion usually limited in extent. Pattern C demonstrates nodular invasive carcinoma extensively involving both the intraluminal preinvasive components and the adjacent bile duct mucosa and wall. IPNBs without invasive carcinoma show more favorable postoperative overall survival (OS) compared with IPNBs with invasion overall and with those exhibiting patterns B and C; however, their postoperative OS is comparable to that observed in pattern A. No significant difference in survival has been reported between patterns B and C [66].

Regarding the microvasculature supplying IPNB and IOPN, approximately half of IPNBs and IOPNs with less complex architecture (type 1) utilize the PCP as supporting vessels through vessel co-option [25], whereas the associated invasive carcinoma is supplied by neoangiogenic vessels accompanied by fibrous stroma. In contrast, the intraluminal components of the remaining IPNB cases with more complex architecture (type 2) exhibit sparse capillaries without fibrous stroma, representing a pattern distinct from both vessel co-option and neoangiogenesis [25].

**Molecular and genetic alterations**

***(1) IPNB***

***(a) Molecular and genetic alterations***

The frequencies of molecular and genetic alterations in IPNB may vary according to geographic region, and intertumoral heterogeneity is considerable, potentially reflecting subtype, anatomical location, and tumor progression [10,59,67,68,69,70]. Recent studies from Taiwan and Germany have identified frequent mutations in *KRAS*, *SMAD4*, *TP53*, and *ERBB2*, as well as mutations in *CDKN2A*, *GNAS*, *RNF43*, *APC*, and *CTNNB1*, which play pivotal roles in IPNB tumorigenesis. These findings suggest that IPNB is a heterogeneous disease characterized by multiple driver mutations. The genetic alterations observed in IPNB share similarities with those of LD-iCCA, p/d-CCA and pancreatic carcinoma, whereas genetic profiles characteristic of SD-iCCA have not been reported in IPNB.

For example, in the gastric subtype, mutations have been detected in *KRAS* (60%), *STK11* (40%), *KMT2C* (40%), *APC* (20%), *CTNNB1* (13%), and *TP53* (13%) [68]. Immunohistochemically, aberrant STK11 expression appears to be specific to the gastric subtype [68]. *KRAS* mutations are predominantly identified in preinvasive lesions, supporting their role as early events in gastric subtype tumorigenesis. *TP53* and *PIK3CA* mutations are frequently detected in extrahepatic intestinal-type IPNBs, whereas *KRAS* and *GNAS* mutations are more common in intrahepatic intestinal-type IPNBs [59], suggesting anatomical differences in tumorigenesis along the biliary tree. Identical mutations have been consistently observed in both preinvasive lesions and corresponding invasive carcinomas in the gastric subtype, supporting clonal progression. *KRAS* mutations are enriched in intrahepatic IPNB (42%), whereas *SMAD4* mutations are more frequently detected in extrahepatic IPNB (21%). Mutational signature analysis has revealed that *SBS1* and *SBS5* signatures are homogeneously enriched in intrahepatic IPNB [67,68].

The accumulation of genetic alterations in IPNB may contribute to the early phase of stromal invasion [66,67,68,69]. Copy number aberrations increase progressively from low-grade to high-grade IPNB and eventually to invasive carcinoma. Furthermore, multifocal independent carcinogenic events have been observed in IPNB, resulting in mutationally distinct carcinoma lesions. *STK11* mutations have been detected exclusively in invasive and lymph node metastatic cases but not in high-grade dysplasia cases [68].

***(b) Type 1 and 2 and genetic changes***

Mutations in *KRAS* and *GNAS* are enriched in type 1 IPNB, whereas mutations in *TP53*, *SMAD4*, and *KMT2C* are enriched in type 2 IPNB [70]. Recently, Doi et al. reported that DNMT1 protein expression was significantly higher (*p* < 0.001) in 28.6% of type 1 cases and in all type 2 cases [63]. The overall DNA methylation ratio across six examined genes, including SOX17, was lower in type 1 than in type 2 (*p* < 0.05 for each comparison). Type 2 IPNB exhibited increased DNMT1 expression and a higher frequency of DNA methylation in the analyzed tumor suppressor genes compared with type 1 [63].

***(2) IOPN***

All biliary IOPNs have been found to harbor recurrent gene fusions involving *PRKACA* or *PRKACB* [40,60]. These fusions have also been identified in cholangiocarcinomas derived from IOPN, as well as in matched bile duct brushing specimens. Such gene rearrangements were absent in all 126 control pancreatobiliary lesions examined. Pancreatic IOPN appears to be virtually identical to biliary IOPN in clinicopathological characteristics and molecular background, including specific genetic alterations involving *PRKACA* [40,60]. These findings support the concept that IOPN represents a molecularly distinct subtype of neoplasm within the biliary and pancreatic systems.

#### 3.1.3. Intraductal Tubulopapillary Neoplasm of the Bile Duct (Biliary ITPN)

Biliary ITPN (bITPN) has been reported to share pathological and molecular features with pancreatic ITPN (pITPN) [11,12,13,71,72,73,74,75], which is recognized as a precursor of PDAC [1,71,72,73,74,75]. Accordingly, bITPN has also been regarded as a precursor lesion of CCA [11,12,13,74,75]. Herein, pITPN is first discussed briefly.

**Pathology of pancreatic ITPN (pITPN)**

pITPN forms solid, fleshy to rubbery, single or multiple nodular masses within dilated pancreatic ducts [1,71,72,73,74]. These gross features suggest expansile intraductal tumors growing and continuously spreading within the dilated main and branch pancreatic ducts. Histologically, pITPN demonstrates predominantly back-to-back non-mucinous tubular glands with minimal or no intervening stroma, often resulting in cribriform structures [1,71,72,73,74,75]. pITPN typically exhibits high-grade dysplasia or carcinoma in situ throughout the lesion, without areas of low-grade atypia [1]. Approximately 30–70% of cases are associated with invasive carcinoma [71,72,73,74,75]. The invasive component is usually limited in extent and typically shows a solid tubulopapillary pattern identical to that of the intraductal component, differing from conventional tubular adenocarcinoma.

Immunohistochemically, the neoplastic cells are consistently positive for pancytokeratin, including CK7 and CK19, EMA, and MUC1; MUC6 is commonly expressed, whereas MUC5AC—a marker of all types of IPMN—is not expressed in pITPN. No mutations have been detected in *KRAS* or *BRAF* [71,72,73,74,75].

**Pathology of bITPN**

A similar, if not identical, tumor to pITPN is known to develop within the lumen of the bile duct and is designated bITPN [11,12,13]. bITPN presents as grossly visible, cast-like nodular tumors, most commonly found in the lumen of intrahepatic bile ducts and occasionally in perihilar and distal bile ducts [11,12,13]. Tumor size ranges from 0.6 to 8 cm (mean, 6.9 cm) [11,12,13], and the neoplastic nodules often fill the bile duct lumen [11,12,13]. Intrahepatic bITPNs tend to be more nodular, and a striking snake-like intraductal growth pattern has been described [11,12,13].

**Figure 3:**
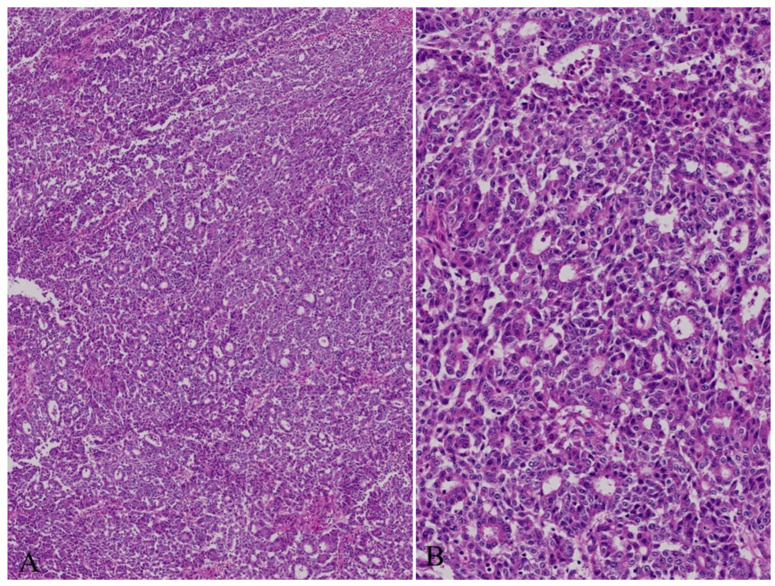
Intraductal tubulopapillary neoplasm of the bile duct (ITPN). (**A**): Intraductal neoplasm in the hilar bile duct showing a predominantly tubular pattern with cribriform architecture. ×100. Hematoxylin and eosin (H&E) staining. (**B**): The neoplasm is composed of highly dysplastic epithelium forming tubular and cribriform patterns. Higher magnification of (**A**). ×150. H&E staining.

Histologically, bITPN is composed of non-mucinous cuboidal to low-columnar epithelium forming back-to-back tubular or tubulopapillary structures, as well as cribriform or solid patterns, usually with minimal or no intervening stroma (Figure 3A,B) [11,12,13]. Virtually all bITPNs are regarded as high-grade dysplasia or carcinoma in situ, similar to pITPN. Tumor necrosis is observed in approximately 85% of bITPN cases, and central necrosis resembling mammary-type comedonecrosis is identified in approximately 40% of cases.

**Molecular and genetic features**

Immunohistochemically, MUC1 is expressed in approximately 80% of bITPN cases, and most bITPNs show variable MUC6 expression, whereas MUC5AC and MUC2 are not expressed [11,12,13,73,74,75]. Compared with IPNB, ITPN exhibits fewer genetic mutations [11]. The molecular and genetic profile of bITPN is characterized by frequent alterations in cell cycle–related and chromatin remodeling genes, which are generally absent in IPNB and IOPN [11,12,13]. Reported molecular alterations include *CDKN2A/p16* abnormalities (intraductal component: 44%; invasive component: 33%) and *TP53* mutations (intraductal component: 17%; invasive component: 9%), whereas *KRAS* and *PIK3CA* mutations and loss of *SMAD4* are rare.

**Invasion and spread**

Approximately 20% of bITPNs are confined to the bile duct lumen (pTis) [11,12,13], whereas the remaining 80% are associated with stromal invasion. Notably, approximately 50% of invasive cases demonstrate nodular sclerosing invasion into the hepatic parenchyma, resembling SD-iCCA [11,12,13]. Indeed, some invasive carcinomas arising from bITPN have been classified as SD-iCCA and others as LD-iCCA [75]. Foci of invasion are typically represented by firm, scirrhous nodular lesions with irregular borders; however, these lesions are often relatively well demarcated from the surrounding hepatic parenchyma [12]. Several distinct patterns of invasion have been described [11,12,13].

***(i) Infiltrating tubular carcinoma indistinguishable from conventional tubular CCA:*** Schlitter et al. [12] reported that most invasive carcinomas showed either focal or predominant tubular patterns composed of relatively small tubular units within desmoplastic stroma [11,12,13], rendering them virtually indistinguishable from conventional tubular adenocarcinoma. In approximately 50% of invasive bITPN cases, this tubular invasive pattern was predominant.

***(ii) “Invasive carcinoma” mimicking in situ-like carcinoma:*** Approximately one fourth of invasive bITPNs exhibit so-called invasive carcinoma that mimics in situ-like carcinoma or preinvasive tumors and displays an expansile growth pattern, typically with central comedo-necrosis, resembling the main intraductal component of bITPN [12]. This pattern is highly unusual and may be relatively specific to bITPN. It appears analogous to the distinctive invasive pattern described in pITPN but differs from that observed in conventional CCA. At least some of these neoplastic nodules may represent intraductal spread from bITPN—namely, intraepithelial involvement of adjacent bile ducts surrounding the main bITPN tumor—rather than true stromal invasion [11,12,13].

***(iii) Tubulocystic formation with large cystic units:*** In approximately 13% of bITPN cases [12], the invasive component is composed of large cystic units forming a deceptively circumscribed lesion with a sieve-like pattern characteristic of tubulocystic carcinoma of the bile duct [76]. In these areas, the cysts are lined by attenuated, bland-appearing epithelial cells, and in some cysts, abortive daughter nests with a tubulopapillary pattern can be observed [12].

**Could bITPN be heterogeneous?**

The pathological heterogeneity of the intraductal components, together with the distinctive features of the associated invasive carcinoma and intra-biliary spreading, suggests that bITPN may not represent a single biliary neoplasm but rather a group of heterogeneous neoplasms. At least two variants have been proposed (Table 3) [7,12,44].

***(1) bITPN with cystic changes***

Schlitter et al. [12] reported that some bITPNs exhibited invasive components resembling tubulocystic carcinoma of the bile duct [76], with large cystic units forming a sieve-like pattern and intratubular nests showing focal transition to more conventional bITPN in the noninvasive component [12]. The latter were characterized by conglomerates of cyst-forming ducts and abortive daughter nests with tubular or tubulopapillary architecture [12]. The case reported by Park et al. [43] similarly demonstrated intraductal tumorous lesions compatible with bITPN associated with conglomerates of microcystic lesions containing papillary or papillotubular components.

Zen et al. described two patients with distinctive biliary cystic tumors [44]. One patient presented with a partially cystic mass in the hepatic hilum, whereas the other had multiple hilar cysts, some of which were obliterated by papillary nodules. Histologically, both tumors consisted of intracystic noninvasive, well-differentiated adenocarcinoma with papillotubular architecture. The associated cysts in these cases may have represented peribiliary cysts [77] and were partially lined by carcinoma cells continuous with the intracystic papillotubular masses. Both tumors shared an identical immunophenotype: CK7(+)/CK20(−)/MUC1(+)/MUC2(−)/MUC5AC(−)/MUC6(+), similar to that of peribiliary glands [20,24]. KRAS and BRAF showed wild-type genotypes in these cases. These pathological and genetic features resemble those of pITPN [71,72,73,74,75], suggesting that bITPN can develop in association with peribiliary glands and cysts [44]. Sato et al. also reported similar papillary and microcystic biliary neoplasms arising in peribiliary glands [78].

Accordingly, it is plausible that at least some bITPNs with large cystic units forming a sieve-like pattern [12], which share features with the cystic and papillary tumors reported by Zen et al. [44] and Park et al. [43], may be related to peribiliary glands and their cystic alterations during tumor development, as first suggested by Schlitter et al. [12].

***(2) bITPN associated with mass-forming SD-iCCA***

**Figure 4:**
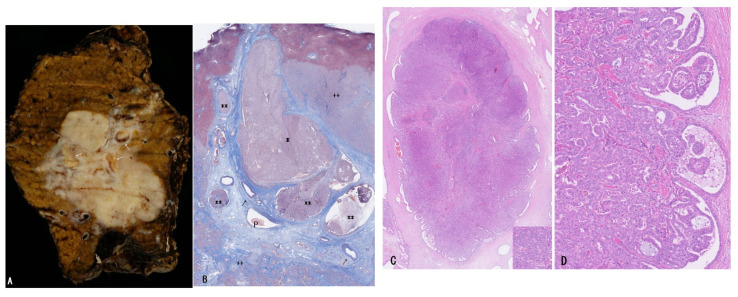
Intraductal tubulopapillary neoplasm of the bile duct (ITPN). (**A**): Cast-like tumors in a dilated intrahepatic bile duct (arrows) in mass-forming intrahepatic cholangiocarcinoma. (**B**): Neoplasms grow in a cast-like pattern within the dilated large bile duct (*) and in adjacent bile ducts (**). “++” indicates invasive nodular carcinoma in the hepatic parenchyma. Hepatic arterial branch (arrows). ×5. Azan–Mallory staining. (**C**): A cast-like tumor impacted within a large bile duct is composed of solid and back-to-back tubular neoplastic units with slit-like spaces at the periphery. No stromal invasion is identified in this lesion. Inset: higher magnification of the central area showing back-to-back tubular structures. ×15. Hematoxylin and eosin (H&E) staining. (**D**): Back-to-back tubular structures with slit-like lumina and abortive papillary patterns are present at the periphery. ×100. H&E staining.

Recently, two cases of bITPN associated with mass-forming SD-iCCA were reported [7]. In these cases, neoplastic nodules with an in situ-like appearance were regionally clustered within and at the periphery of the mass-forming invasive carcinoma and were categorized into larger and medium-to-smaller nodules. The larger neoplastic nodules consisted of high-grade back-to-back tubular and/or solid non-mucinous neoplasms impacted within dilated intrahepatic large bile ducts (6 mm and 10 mm in short diameter), and their gross and microscopic features were identical to those of bITPN (Figure 4A–D). These nodules were negative for MUC5AC and MUC2 but variably positive for MUC6, consistent with the characteristic immunophenotype of bITPN [11,12,13]. The surrounding peribiliary glands were neither neoplastic nor cystically dilated. In addition, around or contiguous with these bITPN lesions in the large bile ducts, multiple medium-to-smaller nodules (200 μm to 2 mm in short diameter) exhibiting tubulopapillary and cribriform patterns were regionally identified. These nodules closely resembled the previously described “invasive carcinoma” mimicking in situ-like carcinoma in bITPN [12] and were also similar to COD or bile duct tumor thrombus of SD-iCCA [3,6]. Interestingly, these intraductal spreading features also closely resemble the intraductal spread and invasive patterns described in pITPN [1,71,72].

Accordingly, bITPN with “invasive carcinoma” mimicking in situ-like carcinoma, as well as nodular invasive carcinoma resembling SD-iCCA [11,12,13], may correspond to or be closely related to this variant [7]. This observation raises the possibility that some intrahepatic ITPNs may arise in close association with SD-iCCA. Recently, Goeppert et al. reported that two cases of intrahepatic ITPN associated with SD-iCCA harbored either an *IDH1* mutation or an *FGFR2* mutation, both of which are characteristic of SD-iCCA [75], further supporting this hypothesis. However, it remains controversial whether such bITPNs represent true primary bITPN or so-called cancerization of the large duct by SD-iCCA, particularly in tumors positive for MUC6 but negative for MUC5AC.

**Cells of origin of bITPN**

As discussed above, one variant of bITPN exhibits large cystic units forming sieve-like and papillary or papillotubular patterns [12] and may be related to peribiliary glands or peribiliary cysts in its development [12,44]. Notably, these tumors share the immunophenotypic profile of peribiliary glands [24]. Accordingly, it is plausible that a precursor lesion may arise within peribiliary glands or peribiliary cysts [12,44], subsequently spreading and proliferating intraepithelially and extending into the lumen of adjacent large bile ducts through their own conduits, ultimately resulting in the development of bITPN with cystic units forming a sieve-like pattern [12].

Second, a precursor of bITPN may also initially arise in bile ductules or smaller bile ducts [7]. Such neoplasms may undergo malignant transformation and invade the hepatic parenchyma, resulting in mass-forming or nodular sclerosing invasive carcinoma consistent with SD-iCCA. Concurrently, neoplastic cells may spread intraluminally and continuously along adjacent small- to medium-sized ducts and eventually into larger bile ducts, giving rise to an intraductal neoplasm that fulfills the diagnostic criteria of bITPN [7]. As noted above, some intrahepatic bITPNs have demonstrated genetic alterations characteristic of SD-iCCA [75], supporting this proposed scenario. Interestingly, our two reported cases [7] additionally exhibited luminal EMA and MUC1 expression and cytoplasmic MUC6 expression but were negative for S100P and MUC5AC; these immunophenotypic features are also characteristic of bile ductules or smaller bile ducts. The invasive components of the nodular or mass-forming carcinomas in these cases differed from LD-iCCA and p/d-CCA, which typically show gross periductal longitudinal infiltrative growth, aggressive behavior with perineural invasion, and positivity for MUC5AC and S100P [5,10]. Moreover, Schlitter et al. reported that approximately 50% of bITPNs were associated with nodular sclerosing invasive carcinoma [12], and Goeppert et al. found that three of five bITPNs were associated with SD-iCCA [75].

In this context, it is plausible that the cells of origin of a majority of so-called bITPNs may be either bile ductules or peribiliary glands and their derived cysts, both of which exhibit immunohistochemical features similar to those of bITPN (Table 3). Notably, hepatic stem cells have been identified in the former, whereas pancreatobiliary stem cells reside in the latter, and these stem cell populations [7,23,36] may be involved in the tumorigenesis of bITPN, respectively.

According to the recently proposed classification scheme for CCA by Liau et al. [79], invasive carcinomas arising in 56% of bITPN cases were categorized as cholangiolar, and 31% were classified as intermediate. Briefly, the cholangiolar and intermediate types of invasive carcinoma arising from bITPN are characterized by architecturally complex growth patterns, including interconnecting small tubules and cribriform structures, along with scant cytoplasm and bland-appearing nuclei [12,13]. Interestingly, bITPNs exhibiting features of the bile duct type consistent with pancreatobiliary adenocarcinoma are rare [79].

Accordingly, it is plausible that bITPN is heterogeneous and that there may be at least two types of cells of origin, as discussed above. Specifically, a majority of bITPNs arising in intrahepatic bile ducts and associated with nodular sclerosing invasion or SD-iCCA [7,12] may originate from bile ductules or small bile ducts, potentially through cancerization of the duct by SD-iCCA [3]. In contrast, some bITPNs—particularly those confined to the lumen of large bile ducts (pTis) and those occurring in perihilar or extrahepatic bile ducts—may arise in association with peribiliary glands or peribiliary cysts. In this context, re-evaluation of previously reported bITPN cases may be warranted [12,43,44], with careful consideration of these two proposed variants and their respective cells of origin.

### 3.2. Secondary Intraepithelial Growth or Spread of Biliary Neoplasms

Biliary neoplasms are known to exhibit several forms of secondary intraepithelial growth or spread within the lumen of the bile ducts in addition to the primary growth at their site of origin. While they may represent a single pathogenetic process such as field change in biliary neoplasm, the following several processes could also be operative.

#### 3.2.1. Continuous Intraepithelial Spread Directly from the Primary Growth Site

A review of recent literature [5,7,10,58,80] indicates that lateral intraepithelial spread directly and continuously from the primary growth site is almost invariably observed, although to varying degrees, in high-grade BilIN, IPNB, and IOPN. Similar intraepithelial spread is also frequently identified in the bile duct mucosa adjacent to COD associated with SD-iCCA [3,7]. These intraepithelial neoplasms spread superficially and laterally along the luminal surface and may also extend vertically into the peribiliary glands and their conduits [80]. Intraductal cast-like extension without stromal invasion has also been reported in some primary biliary neoplasms [3,7]. In this context, the bile duct lumen may serve as an additional route of cancer spread, alongside the bile duct wall, blood vessels, lymphatic vessels, and nerve fibers.

These intraepithelial spreading lesions exhibit atypical nuclear features, including hyperchromasia, pleomorphism, and nuclear irregularities, as well as pseudostratification or micropapillary architecture with loss of polarity, resembling high-grade dysplasia or carcinoma in situ [5,10,58,81]. Their morphological, phenotypic, and immunohistochemical characteristics—including cellular lineage—are similar or identical to those of the primary precursor lesions. These lesions demonstrate either gradual or abrupt transition to adjacent non-neoplastic bile duct epithelium. Immunohistochemical staining for S100P is useful in identifying lateral spread and distinguishing neoplastic epithelium from adjacent non-neoplastic or reactive epithelium [5,10,58]. Such lesions may correspond to high-grade dysplasia or carcinoma in situ identified at the bile duct surgical margin following resection of CCA, IPNB, or IOPN [10,47,48,49].

Because stromal invasion may occur at any site within the affected bile ducts, multiple histologic sections are required to exclude invasive foci [5,10,58].

Chain-like capillaries of the PCP were regularly and densely discernible beneath these laterally spreading intraepithelial neoplasms along the bile duct lumen and vertically within the peribiliary glands. Notably, these capillaries were continuous with the PCP underlying adjacent non-neoplastic bile ducts and peribiliary glands, suggesting vessel co-option of normal bile ducts and peribiliary glands by the intraepithelial neoplastic lesions [25].

Three types of intraepithelial spread have been described.

**Lateral intraepithelial continuous spread of precursor lesions on the luminal surface of the bile duct**

**Figure 5:**
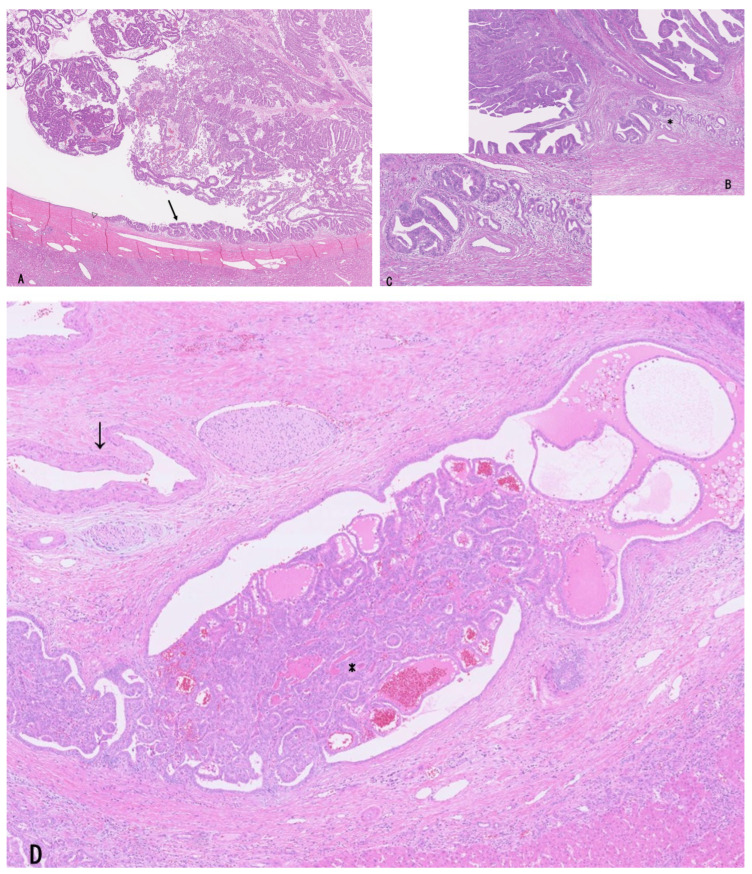
Secondary intraepithelial spreading and growth in the large bile duct. (**A**): Intraductal papillary neoplasm of the bile duct (IPNB). The surrounding mucosa is replaced by intraepithelial neoplasia extending from the primary neoplastic site (arrow), and this lesion shows an abrupt transition to the adjacent non-neoplastic biliary epithelium (△). ×100. Hematoxylin and eosin (H&E) staining. (**B**): At the base of IPNB, peribiliary glands are replaced by intraepithelial neoplasia (*). ×120. H&E staining. (**C**): The left portion of the peribiliary glands is replaced by intraepithelial spreading neoplasm. ×140. Higher magnification of (**B**). ×140. H&E staining. (**D**): Cast-like extension of neoplastic epithelium (*) in biliary intraductal tubulopapillary neoplasm (bITPN). A hepatic arterial branch (arrow) is adjacent to this cast-like extension. ×120. H&E staining.

These lesions exhibit lateral, often extensive, spread of neoplasms such as IPNB, IOPN, and high-grade BilIN [5,10,81]. They may be grossly unrecognizable or may present as rough, finely granular, discolored, short papillary, or multinodular mucosa surrounding the primary neoplastic lesion (Figure 5A) [5,10,58]. In IPNB and IOPN, such intraepithelial spreading neoplasms are frequently identified and may be grossly visible as multifocal papillary, short papillary, or micronodular lesions [5,10,58], resulting in multifocal or multicentric tumors. Consequently, IPNB and IOPN have historically been referred to as biliary papillomatosis [5,10,82]. Although the extent of spread is variable, many cases demonstrate involvement of more than one anatomical segment of the bile ducts, leading to the formation of a sizable mucosal neoplastic lesion. In some cases, the spread may extensively involve both intrahepatic and extrahepatic bile ducts [5,10,58].

Intraepithelial neoplasms extending from high-grade BilIN adjacent to invasive CCA and those observed around IPNB and IOPN are histopathologically and immunohistochemically similar [5,10,58]. However, the latter tend to exhibit more pronounced papillary architecture, whereas the former are more frequently flat or pseudostratified.

**Vertical intraepithelial spread into the peribiliary glands and their conduits**

These continuous intraepithelial neoplasms in high-grade BilIN, IPNB, and IOPN also extend vertically into the peribiliary glands and their conduits, which are located around intrahepatic large bile ducts and p/d-bile ducts (Figure 5B,C) [5,10,58]. In bile ducts affected by invasive CCA, such intraepithelial extension involving the peribiliary glands and the invasive carcinoma itself may be intermingled [5,10,58,80].

**Intraductal cast-like spread**

This type of intraepithelial and intraductal spread has been reported in the main and branch pancreatic ducts in pancreatic ITPN [1,71,72,73]. Similar intraductal cast-like spread can also be encountered in multiple medium-sized and large bile ducts in bITPN (Figure 5D) [3,7,11,12,13], whereas such spread has not been described in other precursors, including high-grade BilIN, IPNB, or IOPN [5,10].

#### 3.2.2. Multifocal Occurrence of Biliary Neoplasm

Metachronous or synchronous multifocal biliary tumors without intervening dysplasia or in situ-like carcinoma between them have occasionally been observed [14,15,83]. Although metastasis via blood vessels or lymphatics cannot be entirely excluded, several pathological mechanisms have been proposed based on the predominant mucosal localization of these neoplasms [14,15,84,85,86,87,88].

**Intrabiliary implantation (tumor seeding)**

Implantation of neoplasms from one site to another within canal organs, such as the urinary or colorectal tract, is well recognized and mms: discontinuous intraepithelial spread or growth account for discontinuous spread of primary neoplasia, resulting in multiple and metachronous tumors [89,90]. The possibility of implantation is supported by the nearly identical morphological features observed among two or more neoplastic lesions [89,90]. The increasing use of transampullary biliary interventions, such as stenting and endoscopic biopsy, may increase the risk of intrabiliary implantation in biliary neoplasms, including CCA, resulting in iatrogenic tumor seeding [14,91,92]. While the exact incidence is not available in the literature, such implantation seems quite low.

***(1) Pathology and molecular mecha*** ***nisms***

In addition to similar or identical histological features, the immunophenotypic profiles of cytokeratins and mucin core proteins were almost identical between the primary and implanted neoplastic lesions, supporting tumor cell seeding from the primary neoplastic site [14]. Identical molecular signatures, including specific gene mutations [14], were also identified in both primary and metachronous biliary neoplasms, further supporting tumor cell implantation [14].

***(2) Biliary neoplasms potentially associated with implantation***

The following biliary neoplasms may be associated with intraductal implantation from the primary neoplastic site.

***(a) IPNB***

IPNB occasionally presents as metachronous or synchronous multiple papillary neoplasms along the bile duct in a multifocal distribution without intervening high-grade dysplasia or carcinoma in situ [5,10,14,15,92]. Some instances of such tumor multiplicity may be explained by intrabiliary implantation of tumor cells [14,93]. Several representative case reports have suggested this mechanism [14,15,93]. Yokode et al. [93] reviewed cases of multiple IPNB and demonstrated that 80% of recurrent, noncontiguous IPNBs developed in the distal bile duct, particularly the common bile duct (CBD) [93]. Whereas 84% of primary IPNBs arise in the intrahepatic or hilar bile ducts, 80% of recurrent IPNBs occurred in the CBD, suggesting that multifocal occurrence was more likely attributable to implantation within the bile duct rather than multicentric tumorigenesis. Furthermore, a case of gastric-type IPNB that developed multifocal recurrence in both intrahepatic and extrahepatic bile ducts following spontaneous detachment of the primary tumor in the CBD during peroral cholangioscopy was reported; the second tumor developed in an upstream segment or on the contralateral side of the biliary tree, raising the possibility of intraluminal implantation in the upstream bile duct [14].

Interestingly, synchronous occurrence of IPNB and IPMN in the same patient has also been reported [94]. Among 10 reported cases of synchronous IPMN and IPNB, most lesions involved the intrahepatic bile duct. Several cases of concomitant IPNB and IPMN with identical genetic and molecular alterations have also been described [95], raising the possibility that one lesion may represent implantation from the other.

***(b) CCA***

Implantation of carcinoma cells or clonal spread of residual cancer cells from the primary tumor via intraluminal dissemination within the bile ducts may occur in CCA following biliary tract manipulation, including endoscopic procedures [14], as reported in IPNB; however, the exact incidence remains uncertain.

***(c) Secondary progression of cancerization of the duct (COD) in the bile ducts by CCA***

Implantation of neoplastic cells may also contribute to the progression of COD in CCA [3]. Specifically, seeding of neoplastic cells detached from previously cancerized bile ducts may promote secondary extension of COD along the bile ducts [3].

***(3) Mechanisms and process of implantation***

Detached neoplastic cells within bile may flow distally and adhere to the mucosal surface—particularly when the tumor has been mechanically manipulated—where they may subsequently proliferate and form a clinically detectable lesion [14,93]. Mechanical tumor seeding during endoscopic procedures, involving iatrogenic dislodgement and dissemination of tumor cells onto damaged mucosa, is also plausible. The increasing use of transampullary biliary interventions, including preoperative stenting and biopsy, may increase the risk of intrabiliary implantation [14,92].

In addition, thick mucinous bile in IPNB and IOPN may facilitate multifocal biliary tumor development by promoting implantation of neoplastic cells [14]. Tumor cell implantation is more likely to occur on damaged mucosal surfaces [90].

**Multicentric tumorigenesis (cancer field)**

True multicentric tumorigenesis may manifest as multiple dysplastic or neoplastic changes within an affected organ, a phenomenon referred to as “field change” or “field cancerization,” particularly in the setting of underlying chronic inflammatory or irritative diseases, such as oral field cancerization [96].

***(1) Multicentric cholangiocarcinogenesis***

Field cancerization involves exposure of the entire biliary epithelium to carcinogenic factors, leading to the development of multiple independent tumors over time [16,97]. Several synchronous or metachronous progressive lesions may arise in the affected bile ducts, resulting in the development of precursor lesions (high-grade dysplasia or carcinoma in situ) and invasive carcinoma.

However, multicentric CCA in the absence of chronic biliary disease or chemical exposure has only occasionally been reported in the bile ducts and gallbladder and appears to be rare in the bile ducts [98,99]. Some earlier reports describing synchronous multiplicity of CCA in the bile ducts documented polypoid or papillary, well-differentiated carcinomas without invasion or with minimal invasion; in retrospect, these lesions may correspond to IPNB or IOPN rather than conventional CCA according to current diagnostic criteria [98,99,100]. As discussed later, multicentric tumorigenesis may also contribute to metachronous recurrence of CCA following curative resection of primary neoplasms [86,87].

***(2) Outbreak of CCA in patients exposed to chemicals***

**Figure 6:**
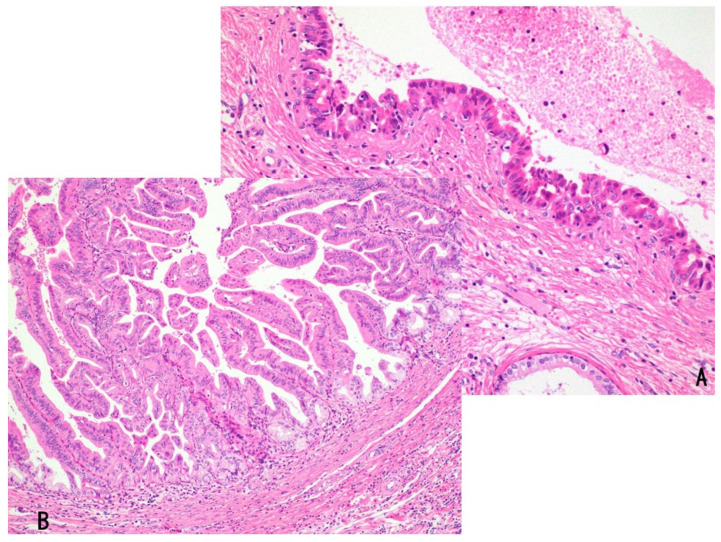
Precursors of cholangiocarcinoma in a patient exposed to chlorinated organic solvents, including dichloromethane and 1,2-dichloropropane. (**A**): Intraductal papillary neoplasm (IPNB)-like lesion. ×150. Hematoxylin and eosin (H&E) staining. (**B**): High-grade biliary intraepithelial neoplasm (BilIN)-like lesion. ×170. H&E staining.

Recently, an outbreak of CCA was reported among workers at a printing company in Japan [85]. These workers had been exposed to chlorinated organic solvents, including dichloromethane and 1,2-dichloropropane [85,101]. This CCA was characterized by precancerous or early carcinomatous lesions at multiple sites involving nearly all large intrahepatic bile ducts, suggesting multicentric tumorigenesis [85,101]. With respect to precursor or early carcinomatous lesions, multiple high-grade BilIN-like lesions as well as IPNB-like lesions were identified (Figure 6A,B). Invasive carcinoma, high-grade BilIN, and IPNB were strongly positive for S100P and γH2AX, whereas non-neoplastic biliary epithelium was negative for S100P and negative or weakly positive for γH2AX. These findings suggest that the carcinogenic process involved chronic bile duct injury and DNA damage affecting nearly all large bile ducts, accompanied by induction of precancerous lesions and progression to invasive carcinoma. Mimaki et al. reported that a substantially higher mutational burden was observed in both invasive carcinomas and precancerous lesions compared with non-occupational CCA [101]. Notably, most identified somatic mutations did not overlap among the lesions, suggesting that these neoplastic lesions were multicentric in origin and that shared mutagenic processes generated distinct somatic mutations at different sites within the bile ducts. These findings imply an increased carcinogenic potential throughout the biliary tree and support the concept of multicentric tumorigenesis.

**Metachronous recurrence of biliary neoplasms**

Metachronous recurrence in the remnant bile duct, defined as the development of a new biliary neoplasm after curative (R0) resection of a primary biliary neoplasm, has occasionally been reported [86,87,102].

***(1) Pathological features***

With respect to their relationship to the primary neoplasm, metachronous tumors have been reported to develop both anterogradely in the distal portion and retrogradely in the proximal portion of the biliary tree [86,87]. A majority of primary and metachronous lesions showed similar histopathological and immunohistochemical profiles, suggesting a shared cellular origin [86,87]. The primary and metachronous neoplasms were spatially separated, indicating that the metachronous lesions were unlikely to represent local recurrence [86,87] or implantation (clonal spread); however, genetic predisposition and multicentric tumorigenesis have been proposed as potential mechanisms underlying metachronous recurrence [86,87,88].

***(2) Genetic alterations in metachronous recurrence of CCA***

The number of genetic mutations was higher in metachronous CCA lesions than in primary CCA lesions, and *CDKN2A* and *SMAD4* were the most frequently mutated genes in metachronous tumors [86,87]. Genetic alterations identified in the primary lesions were also detected in the corresponding metachronous lesions. Specifically, mutations in *CDKN2A*, *AXIN1*, and *APC* were frequently observed in both primary and metachronous tumors. Accumulation of additional genetic alterations beyond those detected in the primary tumors, possibly in association with environmental exposure, may contribute to the development of metachronous tumors [86]. Omori et al. compared the molecular features of paired primary and metachronous CCAs and reported that 83% of metachronous tumors were clonally associated with the corresponding primary tumors, either through direct succession or phylogenetic relatedness [87]. They concluded that more than 80% of metachronous CCAs developing after primary CCA resection are likely to be molecularly related to their primary tumors, suggesting the presence of pre-existing molecular alterations in the biliary epithelium that predispose to the development of metachronous recurrent tumors [87].

**Cancerization of duct (COD)**

It is well established that invasive carcinomas arising from ductal structures and infiltrating the surrounding tissue can re-invade and colonize pre-existing non-neoplastic ducts, resulting in regional intraepithelial growth within the lumen of these ducts [3,8,103]. This process has been referred to as intraductal spread of invasive carcinoma, intraluminal secondary extension, or COD [8,103,104,105,106]. COD, recently reported in hilar CCA and SD-iCCA, may represent one mechanism of secondary occurrence and spread of CCA within the duct lumen [3,4,16]. It is essential to distinguish COD from other intraepithelial neoplasms, particularly precursor lesions. COD has been reported to be associated with poor postoperative OS [4,8].

***(1)***  ***Hallmarks of COD***

The following three features are commonly used for the practical identification of COD in the pancreatobiliary system [4,8].

***(a) Abrupt transition from highly dysplastic epithelium to normal epithelium***

Intraductal and intraepithelial neoplastic lesions demonstrating an abrupt transition between highly dysplastic neoplastic epithelium and adjacent normal duct epithelium, with complete absence of dysplasia in the remaining ducts, represent a characteristic feature of COD [4,8]. Circumferential duct involvement is also frequently observed in COD.

***(b) Close anatomical proximity of COD to invasive carcinoma***

COD is typically located in close proximity to invasive carcinoma and is usually identified in the peripheral areas of, or immediately adjacent to, invasive carcinoma [4,8,104].

***(c) Histological and phenotypic similarities between COD and invasive carcinoma***

Histological, immunohistochemical, and genetic or molecular features are similar between COD and the adjacent invasive carcinoma. In contrast, differences in these features are observed between COD and precursor (preinvasive) neoplasms involving pre-existing ducts [4,8].

***(2)***  ***COD in invasive PDAC***

Several studies have investigated COD in PDAC [8,103,104].

***(a) Incidence***

Suspected COD has been identified on hematoxylin and eosin (H&E)-stained sections in approximately 70–90% of PDAC cases [8].

***(b) Pathology***

Hutchings et al. reported that, histologically, COD involving non-neoplastic pancreatic ducts was highly atypical and pleomorphic and closely resembled the adjacent invasive PDAC [8]. Although COD can mimic a flat or microscopic precursor lesion, namely high-grade pancreatic intraepithelial neoplasia (PanIN), high-grade PanIN typically exhibits milder and more gradual cytological atypia than COD. Immunohistochemically, COD and the adjacent invasive PDAC demonstrated concordant patterns of p53 and SMAD4 expression in 95% and 100% of cases, respectively. In contrast, such expression was rare in isolated high-grade PanIN. This distinction is helpful in differentiating COD from high-grade PanIN [8,104].

***(3)***  ***COD in hilar CCA***

Infiltration of invasive CCA into pre-existing non-neoplastic bile ducts, followed by intraductal and intraepithelial colonization (COD), has also been reported in hilar CCA [4,16]. Despite considerable histologic overlap between high-grade BilIN and COD, these entities represent distinct processes in tumor progression [4,16].

***(a) Incidence***

Recently, Lee et al. [4] reported that COD mimicking high-grade BilIN was identified in 33% of hilar CCA cases on H&E-stained sections.

***(b) Pathology***

COD in hilar CCA exhibits cytoarchitectural and immunohistochemical characteristics similar to those described for COD in PDAC [4,8]. High-grade BilIN is recognized as a preinvasive lesion of hilar CCA [5,10,45], and COD involving non-neoplastic hilar bile ducts can be distinguished from high-grade BilIN using criteria analogous to those applied for COD in invasive PDAC, including immunohistochemical staining for p53 and SMAD4 [8]. Lee et al. reported that although both COD and high-grade BilIN exhibit dysplastic changes, COD lesions are typically more markedly atypical than precursor lesions [4,8]. COD and the adjacent invasive CCA demonstrated concordant patterns of p53 and SMAD4 expression in 95% and 100% of cases, respectively. In contrast, high-grade BilIN and invasive CCA showed significantly lower concordance rates for p53 and SMAD4 expression. Nevertheless, differentiation of COD from high-grade BilIN may be challenging in certain cases [4].

***(4)***  ***COD in SD-iCCA***

Intraductal neoplasms related to COD have recently been reported in non-neoplastic bile ducts in SD-iCCA [3,16].

***(a) Spectrum of intraductal neoplasms in large bile ducts in SD-iCCA***

Recent studies on SD-iCCA [3,6] have occasionally identified intraductal polypoid neoplasms in non-neoplastic bile ducts adjacent to SD-iCCA. Previously, such lesions were described as the IG type of iCCA in the intrahepatic bile ducts [31,32].

***(b) Incidence***

Our recent study demonstrated that approximately one-tenth of SD-iCCA cases exhibited grossly visible polypoid or cast-like cancerization of the duct involving intrahepatic large bile ducts, simulating grossly visible precursor lesions such as IPNB [3]. Mitsui et al. reported that 20% of SD-iCCA cases showed similar neoplastic lesions, including smaller lesions, within pre-existing non-neoplastic bile ducts [6]. Patients with SD-iCCA and polypoid COD had significantly shorter survival than those without polypoid COD, and their survival rates were comparable to those of patients with LD-iCCA [3,6].

***(c) Polypoid neoplasms***

Such COD exhibited intraductal polypoid, protruding, cast-like growths measuring 3–15 mm in greatest dimension, without mucin hypersecretion, and involving one to several adjacent dilated bile ducts located near or at the periphery of mass-forming SD-iCCA [3]. Histologically, these lesions showed well-differentiated papillary or tubular/cribriform patterns with minimal or absent fibrous stroma and no apparent stromal invasion, closely resembling ITPN or IPNB (Figure 5D) [3,6]. No direct invasion from SD-iCCA into the polypoid COD was observed. The intraductal polypoid neoplasms were histologically and immunohistochemically similar to the adjacent SD-iCCA, and both components were generally of the biliary subtype [3,6]. Some lesions appeared to float within the dilated bile ducts, whereas others showed focal attachment to the luminal surface. An abrupt transition was observed between these polypoid neoplasms and the adjacent normal lining epithelium of the affected bile ducts. Taken together, these findings suggest that such intraductal polypoid neoplasms represent COD in SD-iCCA [3], although Mitsui et al. referred to these intraductal lesions as bile duct tumor thrombi (intraductal polypoid tumor growth) [6].

***(d) Molecular alterations***

Immunohistochemical analyses demonstrated that polypoid COD and the adjacent SD-iCCA exhibited identical phenotypic features, suggesting a shared genetic background [3,6]. Mitsui et al. further performed whole-exome sequencing analysis [6] in cases of SD-iCCA with and without polypoid COD. The genetic landscapes were largely similar between SD-iCCA with and without polypoid COD; however, recurrent mutations in *MUC2* and *MUC17*, as well as *FGFR2* fusion genes, were more frequently observed in polypoid COD–positive SD-iCCA [6].

***(e) Relationship of polypoid COD to bITPN, IOPN, and IPNB***

***(i) Differences from IPNB and IOPN***

There are several differences between polypoid COD and polypoid precursor lesions such as IPNB and IOPN [3,6]. Grossly visible COD lesions are histologically more homogeneous, whereas polypoid precursors typically display histological heterogeneity [5,10,58]. IPNB comprises biliary (5 cases), intestinal (8 cases), gastric (5 cases), and oncocytic (2 cases) subtypes, and approximately half of IPNB cases are noninvasive. This feature contrasts with polypoid COD, which consistently exhibits a biliary phenotype similar to that of the adjacent mass-forming SD-iCCA. Approximately half of IPNB and IOPN cases are noninvasive, and the remaining cases show mild or minimal stromal invasion at the time of diagnosis. In contrast, all cases of polypoid COD were associated with mass-forming invasive CCA, although the polypoid COD lesions themselves were apparently noninvasive [3].

***(ii) Differences from bITPN***

ITPN presents as an intraductal, cast-like, predominantly tubular neoplasm with positive MUC6 and negative MUC5AC expression, occurring mainly in the intrahepatic bile ducts and occasionally in the hilar bile ducts [11,12,13]. It is conceivable that, in a subset of SD-iCCA—particularly those that are MUC5AC-negative and MUC6-positive—cancerized carcinoma cells may grow and spread extensively within smaller and larger bile ducts via intraductal cast-like extension [7], and some may enlarge within large bile ducts to form cast-like intraductal tumors. Such COD lesions may closely resemble bITPN, making differentiation between polypoid COD and bITPN challenging. In this context, COD has been proposed as a potential subtype or mimic of bITPN [3,7].

***(iii) Differentiation from polypoid invasive CCA***

Polypoid COD associated with SD-iCCA differs from polypoid invasive CCA, which typically exhibits the histological features of LD-iCCA or p/d-CCA and demonstrates invasive growth continuous with adjacent or surrounding periductal infiltrating carcinoma [17] (see below).

### 3.3. Intraductal Polypoid Invasive Carcinoma

The majority of LD-iCCA and p/d-CCA predominantly infiltrate the bile duct wall and periductal tissue longitudinally and may additionally show nodular invasive growth within the hepatic parenchyma [1,5,10,58]. Although some invasive CCAs exhibit mild intraluminal growth or protrusion into the affected bile ducts, a small subset presents prominent polypoid or cast-like growth within the bile duct lumen that is continuous with periductal infiltrating carcinoma [17], apparently resembling intraductal tumorous or polypoid precursor lesions. Other rare carcinomas also show such growth pattern. While they might have been collectively classified as the IG type or combined periductal infiltrating plus intraductal growth (PI+IG) type according to the macroscopic classification [31,32], this may be composed of several neoplasms.

#### 3.3.1. Polypoid Invasive Carcinoma (PICA) of the Bile Duct

Recently, Taskin et al. [107] proposed the pathologic term “PICA” defined as a neoplastic polyp of the gallbladder distinct from other intracholecystic polypoid neoplasms, including intracholecystic papillary neoplasm (ICPN) and pyloric gland adenoma [1,2]. Notably, PICA lacks a preinvasive or adenomatous component within the polypoid lesion, and the polypoid lesion itself is composed entirely of invasive adenocarcinoma [17,107].

**Figure 7:**
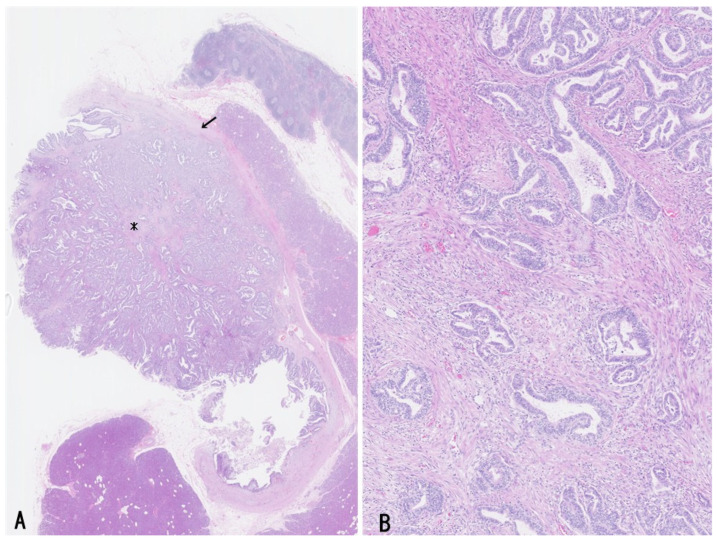
Polypoid invasive cholangiocarcinoma (PICA) of the intrahepatic large bile duct. (**A**): An intraductal polypoid neoplasm (*) is continuous with ductal and periductal infiltrating carcinoma (arrow). ×20. Hematoxylin and eosin (H&E) staining. (**B**): The intraductal polypoid component consists of invasive carcinoma with a desmoplastic reaction and lacks low-grade dysplastic lesions. Higher magnification of (**A**). ×160. H&E staining.

PICA of the bile duct (Figure 7A,B) is rare but has been described, and may be a variant of conventional LD-iCCA and p/dCCA [17]. Histologically, PICA of the bile duct resembles PICA of the gallbladder [107]. Grossly, it presents as a single, sessile, polypoid mass, and the polypoid component consists of invasive carcinoma with papillary or tubular architecture accompanied by active desmoplasia, continuous with infiltrating carcinoma in the bile duct wall and periductal tissue [17].

Interestingly, two of our four PICA cases were associated with nodular sclerosing CCA composed of well- to moderately differentiated adenocarcinoma located in another part of the biliary tract and discontinuous from the PICA lesion. This finding suggests that intrabiliary implantation or multicentric carcinogenesis may have contributed to the development of PICA in these two cases.

#### 3.3.2. Differentiation of PICA from IPNB or IOPN Associated with Invasion

The polypoid component of PICA consists of adenocarcinoma composed of papillary or tubular structures, often with poorly differentiated areas and prominent desmoplasia [17,107]. In contrast to IPNB or IOPN, (i) back-to-back epithelial units containing low-grade or adenomatous components, which reflect the multistep carcinogenesis characteristic of IPNB or IOPN, are absent in PICA; and (ii) direct, continuous, and extensive invasion of the polypoid carcinoma into the duct wall and periductal tissue, with destruction of the fibromuscular layer of the duct wall, is consistently observed. Whereas IPNB demonstrates replacement growth of non-neoplastic peribiliary glands within the bile duct wall by neoplastic cells [5,10,58], such glandular replacement is not observed in PICA [17]. These findings strongly suggest that invasive CCA infiltrates continuously into the bile duct wall and periductal tissue while simultaneously proliferating within the bile duct lumen, resulting in the formation of PICA, and that PICA does not arise from IPNB or IOPN.

In addition, IPNB was first proposed in 2001 [10,108], whereas PICA was introduced in 2021 [17,107]. Therefore, some cases of PICA might previously have been classified as IPNB associated with invasive adenocarcinoma before 2021.

#### 3.3.3. Rare Malignant Tumors Showing Invasive Polypoid Growth in the Lumen of Bile Ducts and Also Invading the Surrounding Liver

In addition to the above-mentioned PICA of bile ducts, other subtypes of CCA and rare malignant tumors may also present prominent intraductal invasive polypoid growth.

**Enteroblastic cholangiocarcinoma**

Enteroblastic CCA is a rare tumor histologically characterized by neoplastic cells with clear cytoplasm and “blastic,” coarse chromatin. Some cases of enteroblastic CCA show predominant intraluminal growth within large bile ducts [109].

**Undifferentiated carcinoma with osteoclast-like giant cells**

This carcinoma is extremely rare in the hepatobiliary system and may present as an intraductal lesion within a dilated large bile duct. Histologically, the tumor predominantly consists of undifferentiated carcinoma with osteoclast-like giant cells, accompanied by minor foci of adenocarcinoma [110].

### 3.4. Bile Duct Tumor Thrombus (BDTT) of Nonbiliary Neoplasms

HCC and also carcinoma arising from extrahepatobiliary organs metastasize to the liver and present with intrabiliary polypoid growth or BDTT [111,112,113,114,115,116,117,118,119].

#### 3.4.1. BDTT of Hepatocellular Carcinoma (HCC)

HCC with BDTT represents a distinct pathological manifestation of HCC [18,111,112,113,114]. Recent studies have reported that approximately 12.9% of patients with HCC develop BDTT. Pathologically, BDTT most commonly occurs in the background liver affected by nodular cirrhosis secondary to hepatitis B virus or hepatitis C virus infection. Patients with HCC and BDTT typically present with more advanced-stage disease and adverse histological features, including moderately to poorly differentiated carcinoma, higher rates of macrovascular invasion and lymphovascular invasion, and overall poorer differentiation. These patients demonstrate a worse prognosis compared with those with HCC without BDTT who undergo surgical treatment.

In general, BDTT appears as a purple-black, soft thrombus without firm adhesion to the bile duct wall [18]. Two pathological types of BDTT have been described. The first type is composed predominantly of tumor cells and appears yellow-gray after fixation. The second type, referred to as “cancerous thrombosis,” consists of blood clots intermixed with tumor cells and is thought to result from hemorrhagic invasion of the bile duct wall. With respect to the primary HCC lesions associated with BDTT formation, the tumors are typically diffuse or infiltrative, show moderate to poor differentiation, lack a capsule or have only a partial capsule, and exhibit marked invasiveness.

The proposed mechanisms underlying BDTT formation include the following: (1) direct invasion of the bile duct by the primary tumor; (2) intrabiliary dissemination through microvascular or lymphatic spread; and (3) extension via periductal neural pathways [18]. However, direct histopathological evidence of bile duct wall invasion is not consistently documented.

#### 3.4.2. BDTT of Extrahepatobiliary Malignancy

Carcinoma arising from extrahepatobiliary organs may metastasize to the liver and present with intrabiliary polypoid growth or BDTT [115,116,117,118,119]. However, compared with HCC, BDTT of extrahepatobiliary origin is extremely rare [115,119]. In colorectal cancer (CRC), liver metastasis—referred to as colorectal liver metastasis (CRLM)—occurs in approximately 20–50% of patients [116,117,118]. CRLM typically presents as nodular lesions within the hepatic parenchyma [2]. Among 151 patients with CRLM, 21 showed intrahepatic bile duct involvement confirmed histologically, and macroscopic BDTT was occasionally identified, often at the time of surgical resection. In addition to CRLM, other carcinomas have also been reported to present with BDTT. For example, pancreatic acinar cell carcinoma (ACC) has been described as causing BDTT. While differentiation of pancreas-derived carcinoma from bile duct origin carcinoma could be difficult diagnostic problem, bile duct origin may include the finding of an in situ high-grade BilIN.

**Figure 8:**
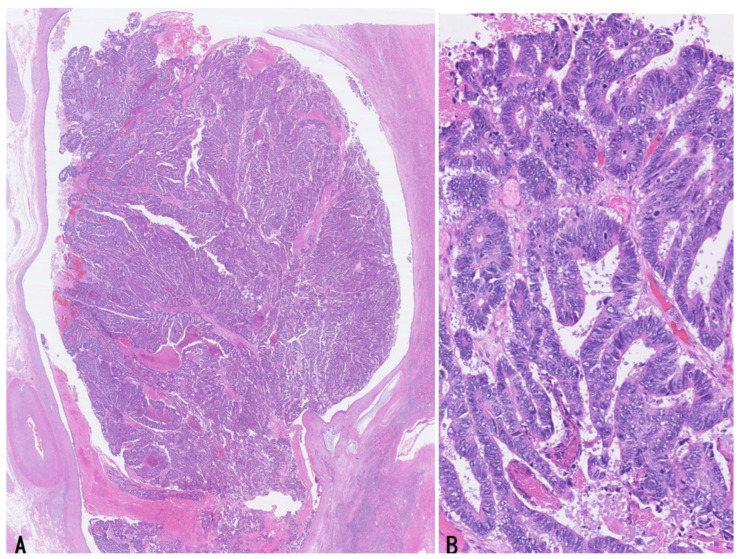
Bile duct tumor thrombus (BDTT) in metastatic colorectal carcinoma to the liver. (**A**): Carcinoma is present within a dilated intrahepatic bile duct. ×100. Hematoxylin and eosin (H&E) staining. (**B**): Higher magnification shows well-differentiated adenocarcinoma with an intestinal phenotype. ×200. H&E staining.

Histopathological examination of primary colorectal cancers (CRC) associated with BDTT revealed moderately to well-differentiated adenocarcinomas [116,117,118,119]. Many patients with macroscopic BDTT exhibit relatively low-grade biological behavior characterized by well-differentiated adenocarcinoma and limited venous invasion (Figure 8A,B). Intraepithelial ductal spread is a common feature in the bile ducts surrounding BDTT in CRLM. Tumors that extend into the bile ducts and develop BDTT are less likely to form prominent parenchymal masses because their growth predominantly occurs within the bile ducts. CRLM with macroscopic BDTT has been reported to confer a better prognosis. However, the relationship between prognosis in CRLM and the presence of BDTT remains controversial.

With respect to the mechanism underlying BDTT formation in CRLM, hepatic metastasis of CRC generally occurs via the portal venous route [115,116]. CRC exhibits a relatively high affinity for bile ducts [116,117,118], and tumor cells metastasize via the portal tracts, invade the biliary epithelium, and proliferate intraepithelially within the bile duct lumen, resulting in the formation of a polypoid tumor or tumor thrombus.

Differentiation of BDTT secondary to CRC from precursors of CCA, particularly intestinal-type IPNB, may occasionally be challenging [120]. A homogeneous intraductal tumor morphology and a history of CRC favor a diagnosis of CRLM with BDTT. CRC extending into the bile ducts should be distinguished from intrahepatic CCA by the absence of dysplastic changes in the surrounding bile duct epithelium and by histological similarity to the primary CRC [121,122]. In addition, immunohistochemical staining for CK7 and CK20 is useful in distinguishing usual SD-iCCA from CRLM with BDTT. A CK20-positive/CK7-negative immunophenotype demonstrates approximately 95% diagnostic accuracy for BDTT and associated intraepithelial spread, whereas a CK7-positive/CK20-negative immunophenotype shows approximately 85% diagnostic accuracy for iCCA and its intraepithelial spread [121].

## 4. Perspective of Intraductal Growing and Spreading Neoplasms

**Figure 9:**
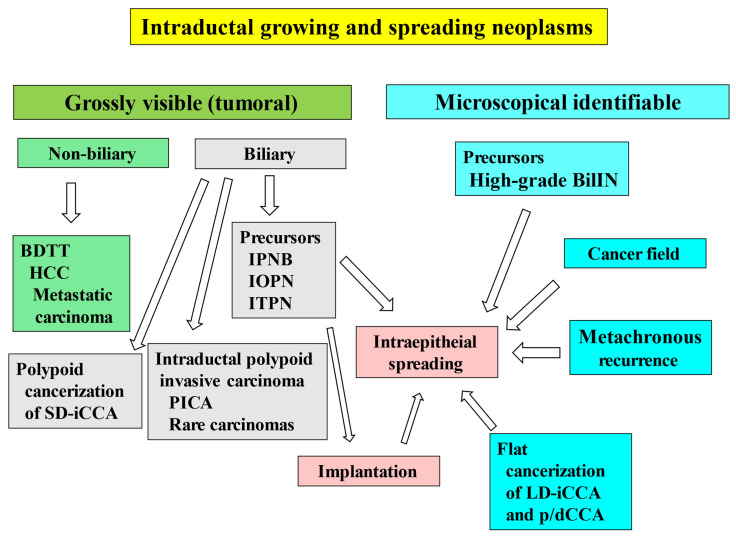
Biliary and nonbiliary neoplasms growing and spreading within the lumen of the bile ducts. They are largely divided into grossly visible (tumoral) and microscopically identifiable lesions. IPNB, intraductal papillary neoplasm of the bile duct; IOPN, intraductal oncocytic papillary neoplasm; ITPN, intraductal tubulopapillary neoplasm; BDTT, bile duct tumor thrombus; HCC, hepatocellular carcinoma; BilIN, biliary intraepithelial neoplasm; CCA, cholangiocarcinoma; SD-iCCA, small-duct-type intrahepatic cholangiocarcinoma; PICA, polypoid invasive carcinoma; LD-iCCA, large-duct-type intrahepatic cholangiocarcinoma; p/dCCA, perihilar and distal cholangiocarcinoma.

Above-mentioned neoplasms growing and spreading in the lumen of the bile ducts are diagrammatically shown in Figure 9. They are not a single disease and are composed of heterogeneous and diverse neoplasms. They can be largely divided into grossly visible (tumoral) and microscopically identifiable neoplasms. The vast majority of them are derived from biliary epithelial cells. Precursors such as high-grade BilIN and IPNB occur in the bile ducts without chronic biliary diseases and also develop in chronic biliary diseases such as primary sclerosing cholangitis and, particularly, liver fluke infection [123,124]. In clinical practice, differential diagnosis is important but challenging. CT and MRI can be used as auxiliary tools for the differentiation of these neoplasms before endoscopic biopsies [125]. Biliary brush cytology is also a challenging approach to diagnosis of these biliary neoplasms [126,127]. Although biliary brush cytology reportedly has a high specificity, its sensitivity is poor. Adding NGS may lead to a significant improvement in the sensitivity [127]. Therapeutic approaches should be based on the relevance of this new categorization of these intraductal neoplasms. For example, chemotherapies for BDTT of HCC [111,112,113,114] could be different from those for precursors of CCA, and molecular targets of polypoid cancerization of SD-iCCA could be different from IPNB and bIOPN arising in the large bile ducts [5,10,40,58].

## 5. Conclusions

Several types of neoplasms can arise, grow, and/or spread within the lumen of intrahepatic large bile ducts and perihilar/distal bile ducts (=large bile ducts), which represent specialized canalicular organs associated with unique peribiliary glands. These neoplasms can be categorized into four groups: (i) precursors of CCA arising in LD-iCCA and perihilar/distal CCA, including high-grade BilIN, IPNB, IOPN, and ITPN; (ii) secondary growth and spread of biliary neoplasms within the bile duct lumen, which can be subdivided into continuous intraepithelial spread of neoplastic epithelium in addition to the primary growth site of precursors, intrabiliary implantation, multicentric tumorigenesis including metachronous recurrence of LD-iCCA and p/d-CCA, and COD associated with invasive CCA; (iii) prominent intraductal polypoid growth of invasive CCA; and (iv) nonbiliary neoplasms, such as hepatocellular carcinoma and extrahepatobiliary malignancies, that mimic primary biliary neoplasms (bile duct tumor thrombus). Intraluminally growing and spreading biliary neoplasms within the large bile ducts do not represent a single disease entity but rather comprise a heterogeneous group. Systematic evaluation of intraluminal growth and spread based on these four categories may facilitate a more comprehensive understanding of biliary tumorigenesis and contribute to the further development of biliary pathology as a distinct field.

## Figures and Tables

**Table 1:** Neoplasms arising, growing, and/or spreading in the lumen of large bile ducts.

A. Precursors of CCA arising in the large bile ducts * (LD-iCCA and p/dCCA) 1. High-grade BilIN 2. IPNB and IOPN 3. ITPN
B. Secondary intraepithelial growth or spread of biliary neoplasms 1. Continuous intraepithelial spread of neoplastic epithelium directly from the primary growth site 2. Multifocal occurrence of biliary neoplasms a. Implantation (tumor seeding) b. Multicentric tumorigenesis (cancer field) c. Metachronous recurrence of biliary neoplasms d. Cancerization of duct (COD) by CCA
C. Intraductal polypoid invasive carcinoma a. Polypoid invasive carcinoma of bile ducts (PICA) b. Rare malignant tumors showing invasive polypoid growth
D. Bile duct tumor thrombus (BDTT) of nonbiliary neoplasms 1. BDTT of hepatocellular carcinoma 2. BDTT of extrahepatic malignant tumors

* Intrahepatic large bile ducts and perihilar and distal bile ducts; CCA, cholangiocarcinoma; LD-iCCA, large-duct-type intrahepatic cholangiocarcinoma; p/dCCA, perihilar and distal cholangiocarcinoma; BilIN, biliary intraepithelial neoplasm; IPNB, intraductal papillary neoplasm of the bile duct; IOPN, intraductal oncocytic papillary neoplasm; ITPN, intraductal tubulopapillary neoplasm.

**Table 2:** Main characteristic features of small intrahepatic bile ducts and bile ductules versus large bile ducts.

Feature	Smaller Bile Ducts and Bile Ductules	Large Bile Ducts *
Location of bile ducts Surrounding connective tissue Small vessels and nerve fibers	Small portal tracts embedded in the hepatic parenchyma Rather dense fibrous tissue Relative sparse	Large biliary tract (larger portal tracts in the liver, at hilar portal tracts and hepatoduodenal ligaments Loose connective tissue containing fatty tissue Relatively dense
Association of peribiliary glands Suspected stem cells	Absent Hepatic stem cells	Frequent Pancreatobiliary stem cells
Peribiliary capillary plexus under lining biliary epithelia	Sparse	Dense and regular
Mucin in the lining epithelia of bile ducts	Negative	Positive
Phenotypes of lining biliary epithelia	Luminal expression of MUC1 and EMA Membranous expression of NCAM and N-cadherin Cytoplasmic expression of CRP and albumin Negative for S100P, MUC3 and MUC5AB	Cytoplasmic and lateral membranous expression of MUC1 and EMA Cytoplasmic expression of S100P, MUC3 and MUC5AB Expression of pancreatic enzymes (pancreatic alpha-amylase, lipase, trypsin/trypsinogen)
Invasive carcinoma Gross pattern	CCA arising in the hepatic paren chyma: SD-iCCA Mass-forming growth pattern	CCA arising in the large bileducts: LD-iCCA, and p/dCCA Periductal infiltrating growth pattern with occasional nodular growth
Precursor lesions	Not identified	High-grade BilIN IPNB IOPN ITPN

* Intrahepatic large bile ducts and perihilar and distal bile ducts; EMA, epithelial membrane antigen; NCAM, neural cell adhesion molecule: CRP, C-reactive protein; CCA, cholangiocarcinoma; LD-iCCA, large-duct-type intrahepatic cholangiocarcinoma; p/dCCA, perihilar and distal cholangiocarcinoma; SD-iCCA, small-duct-type intrahepatic cholangiocarcinoma;BilIN, biliary intraepithelial neoplasm; IPNB, intraductal papillary neoplasm of the bile duct; IOPN, intraductal oncocytic papillary neoplasm; ITPN, intraductal tubulopapillary neoplasm.

**Table 3:** Two possible variants of biliary intraductal tubulopapillary neoplasm (bITPN) with reference to pathologic features and the cell of origin.

Feature	bITPN Associated with Nodular Invasive Carcinoma, Resembling SD-iCCA	bITPN with Cystic/Sheeve-like Changes
Pathologic features	bITPN developing in intrahepatic bile ducts Some cases show genetic alterations detected in SD-iCCA In situ-like carcinoma in adjacent bile ducts resembling bITPN	bITPN arising in perihilar or hilar regions Some cases show genetic alterations detected in LD-iCCA bITPN with cystic, papillary, and tubular architectural patterns
Suspected cell of origin	Bile ductules or small bile ducts	Peribiliary glands and/or derived cysts

bITPN, biliary intraductal tubulopapillary neoplasm; SD-iCCA, small-duct-type intrahepatic cholangiocarcinoma; LD-iCCA, large-duct-type intrahepatic cholangiocarcinoma.

## Data Availability

No new data were created or analyzed in this study.
